# Augmenting Electroencephalogram Transformer for Steady-State Visually Evoked Potential-Based Brain–Computer Interfaces

**DOI:** 10.34133/cbsystems.0379

**Published:** 2025-10-07

**Authors:** Jin Yue, Xiaolin Xiao, Kun Wang, Weibo Yi, Tzyy-Ping Jung, Minpeng Xu, Dong Ming

**Affiliations:** ^1^ Academy of Medical Engineering and Translational Medicine, Tianjin University, Tianjin, People’s Republic of China.; ^2^ Haihe Laboratory of Brain-Computer Interaction and Human-Machine Integration, Tianjin, People’s Republic of China.; ^3^ Beijing Institute of Mechanical Equipment, Beijing, People’s Republic of China.; ^4^ Institute for Neural Computation, the Institute of Engineering in Medicine, and the Department of Bioengineering, University of California at San Diego, San Diego, CA, USA.; ^5^ College of Electrical and Computer Engineering, National Yang Ming Chiao Tung University, Hsinchu, Taiwan.

## Abstract

**Objective:** Advancing high-speed steady-state visually evoked potential (SSVEP)-based brain–computer interface (BCI) systems requires effective electroencephalogram (EEG) decoding through deep learning. However, challenges persist due to data sparsity and the unclear neural basis of most augmentation techniques. Furthermore, effective processing of dynamic EEG signals and accommodating augmented data require a more sophisticated model tailored to the unique characteristics of EEG signals. **Approach:** This study introduces background EEG mixing (BGMix), a novel data augmentation technique grounded in neural principles that enhances training samples by replacing background noise between different classes. Building on this, we propose the augment EEG Transformer (AETF), a Transformer-based model designed to capture the temporal, spatial, and frequential features of EEG signals, leveraging the advantages of Transformer architectures. **Main results:** Experimental evaluations of 2 publicly available SSVEP datasets show the efficacy of the BGMix strategy and the AETF model. The BGMix approach notably improved the average classification accuracy of 4 distinct deep learning models, with increases ranging from 11.06% to 21.39% and 4.81% to 25.17% in the respective datasets. Furthermore, the AETF model outperformed state-of-the-art baseline models, excelling with short training data lengths and achieving the highest information transfer rates (ITRs) of 205.82 ± 15.81 bits/min and 240.03 ± 14.91 bits/min on the 2 datasets. **Significance:** This study introduces a novel EEG augmentation method and a new approach to designing deep learning models informed by the neural processes of EEG. These innovations significantly improve the performance and practicality of high-speed SSVEP-based BCI systems.

## Introduction

Brain–computer interfaces (BCIs) enable the brain to communicate directly with external devices, eliminating the need for peripheral nerves and muscles [1]. This technology finds widespread application across diverse domains, including brain speller [2], machine control [3], rehabilitation [4], emotion recognition [5], and glaucoma detection [6]. Among the various brain signals, the electroencephalogram (EEG) stands out as a preferred choice for constructing BCI systems owing to its affordability, noninvasiveness, and high temporal resolution. Commonly used control signals in BCI systems include P300 [7], motor imaginary (MI) [8], and steady-state visually evoked potential (SSVEP) [9]. Among these paradigms, SSVEP has attracted considerable attention due to its high information transfer rate (ITR) and minimal training requirements, which reflects neural oscillations in the visual cortex elicited by visual stimuli flickering at specific frequencies.

In recent years, researchers have proposed various deep learning models to enhance the efficiency of EEG signal decoding in BCI systems [10]. Models such as ShallowConvNet, DeepConvNet [11], and EEGNet [12] have demonstrated promising results in a range of EEG classification tasks. For SSVEP decoding, specifically, models like Conv-CA [13], FTN, and DTN [14] have been proposed. However, their performance advantages over traditional decomposition methods remain inconclusive in SSVEP classification tasks.

A key challenge for deep learning models in EEG decoding is addressing the issue of data sparsity. These models typically require large datasets to avoid overfitting and achieve robust generalization [15]. However, acquiring substantial amounts of EEG data poses significant challenges and costs because of practical constraints, including complex experiment settings, lengthy experiment time, collection sessions, and limited subject availability [16]. Furthermore, the inherent variability across subjects and datasets exacerbates the problem of EEG data sparsity.

To alleviate such issue, researchers have employed data augmentation techniques for EEG signals [17], including sliding window [18], noise injection [19], and generative adversarial networks [20]. Among these methods, data mixing has gained attention as a straightforward yet effective strategy for generating new samples and improving classification performance. Mixup [21], a foundational data mixing method, combines 2 samples from different classes to create augmented data and has shown success in EEG tasks. For instance, Alwasiti and Yusoff [22] applied the Mixup strategy to a motor imagery classification task, significantly boosting the performance of a convolutional neural network (CNN) model. Similarly, Chen et al. [23] developed a Mixup strategy that integrates EEG signals in the frequency domain. Zoumpourlis and Patras [24] introduced CovMix, which aligns and augments EEG data by combining covariance matrices of EEG signals. These studies demonstrate the potential of data mixing techniques in mitigating the EEG data sparsity. However, most existing approaches that fail to fully utilize strategies do not fully leverage the prior knowledge inherent in EEG signals, which differ fundamentally from image data. Basic data mixing approaches may not align well with the neural basis of EEG signals, necessitating more informed and biologically grounded augmentation strategies.

To effectively handle the increased volume of augmented data and accurately process dynamic EEG signals, models must exhibit greater complexity and be specifically designed to capture the unique characteristics of EEG data. Many existing methods use basic architectures, where the convolution and pooling operations in CNNs often result in the loss of critical temporal information. In contrast, the Transformer architecture [25], renowned for its attention mechanism, excels at preserving temporal information while extracting essential features. Transformers have shown superior performance in processing long sequence data, consequently achieving state-of-the-art results in computer vision (CV) [26], natural language processing (NLP) [27], and various other tasks. In EEG signal processing, several recent studies have explored the application of Transformers to EEG data [28], and most focused on MI classification tasks. For SSVEP classification tasks, existing research studies on Transformer-based models (TF models) are in the primary stage [29,30]. The design and application of TF models for high-speed SSVEP-BCI systems remain largely unexplored, presenting a promising avenue for future investigation.

To address these challenges, this study first introduces a novel data augmentation strategy called background EEG mixing (BGMix). Inspired by the distinct characteristics of EEG components, BGMix combines task-related components with background EEG from different classes to generate valid EEG samples. Alongside BGMix, we propose the augment EEG Transformer (AETF), a TF model that aims to enhance the decoding efficiency of SSVEP-BCI systems. The AETF architecture incorporates a fully connected layer for spatial filtering, a convolutional layer for frequency filtering, and a 2-layer Transformer encoder for temporal feature extraction. This design integrates multidomain features while preserving critical information. The proposed techniques undergo validation through offline experiments using 2 public SSVEP datasets.

## Materials and Methods

### Related work

#### Data mixing strategy

**Fig. 1.**
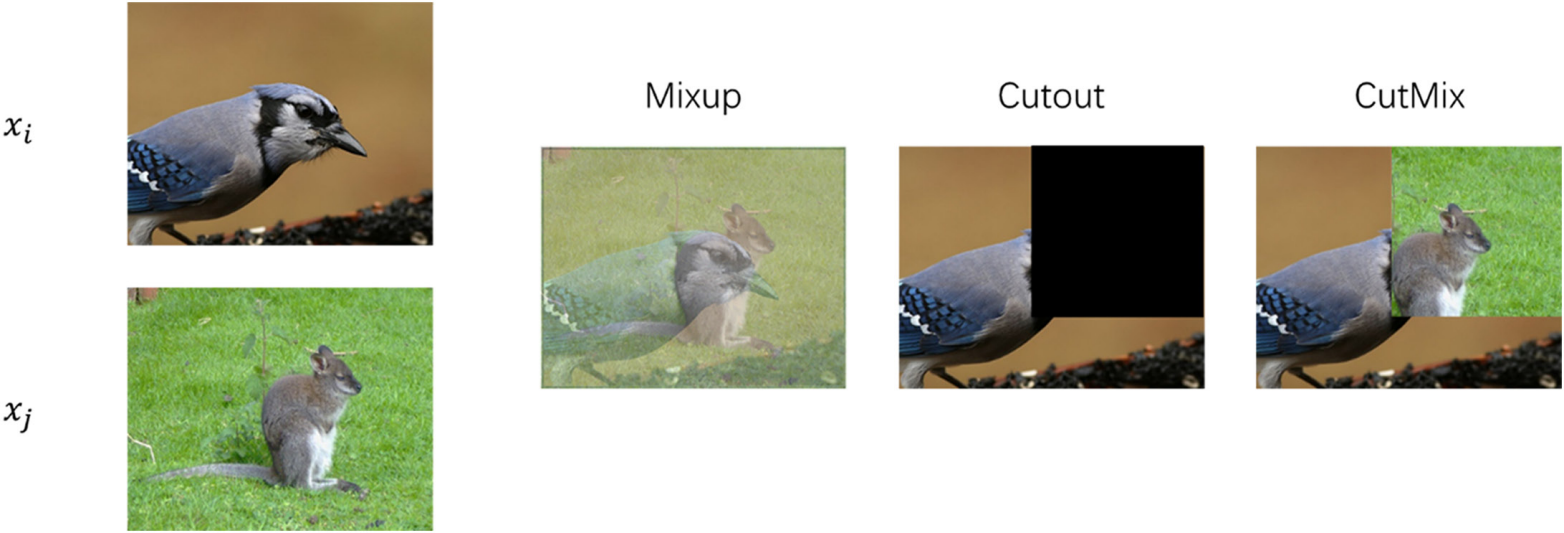
Example of the generated sample of Mixup, Cutout, and CutMix strategies. Images are selected from the ImageNet dataset [61].

In this section, we present 2 data mixing strategies to elucidate their underlying principles. The fundamental concept behind data mixing strategies involves combining samples from distinct classes to create new samples. The first proposed data mixing strategy is known as Mixup [21], originally devised to augment image data. The Mixup strategy could be described as follows:

$$\tilde{x}=\lambda \cdot x_{i}+\left(1-\lambda \right)\cdot x_{j}$$ (1)

$$\tilde{y}=\lambda \cdot y_{i}+\left(1-\lambda \right)\cdot y_{j}$$ (2)

where $x_{i}$ and $x_{j}$ represent 2 samples with different labels $y_{i}$ and $y_{j}$, randomly chosen from the training data, and λ∈[0,1] is sampled from the Beta distribution. To mitigate the problem of unnatural and ambiguous samples encountered in Mixup, the CutMix technique is introduced, which integrates Mixup with the Cutout method [31]. CutMix replaces specific regions of 2 training samples instead of simply combining them. The CutMix is described as follows:

$$\tilde{x}=M⊙x_{i}+\left(1-M\right)⊙x_{j}$$ (3)

$$\tilde{y}=\lambda \cdot y_{i}+\left(1-\lambda \right)\cdot y_{j}$$ (4)

where ⊙ is element-wise multiplication and $M\in {\left[0,1\right]}^{W\times H}$ denotes the specific region to be replaced. A clear example of how these strategies work appears in Fig. 1.

#### Transformer

**Fig. 2.**
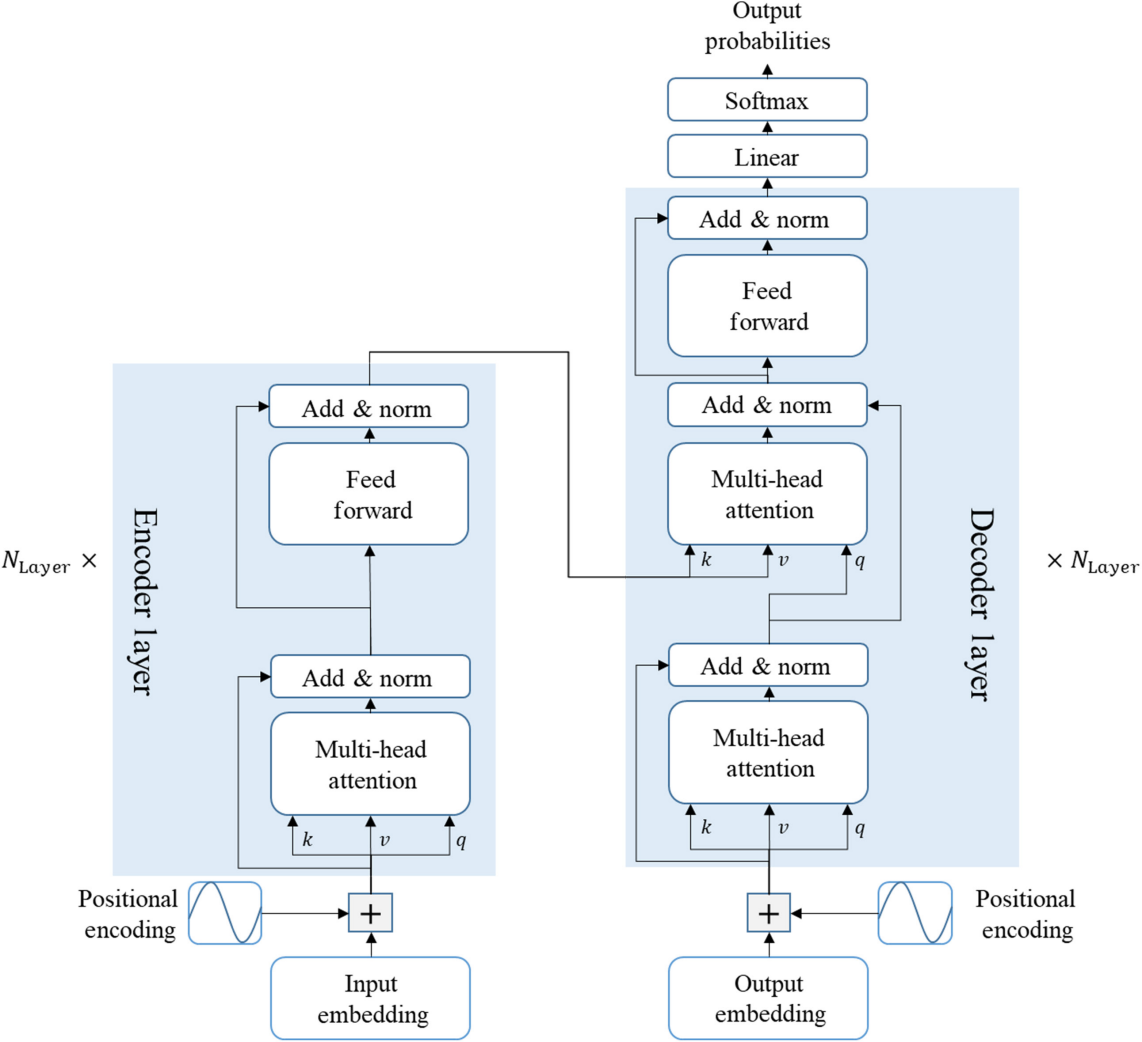
The network architecture of the Transformer. The left part is the encoder, and the right part is the decoder.

In this study, we integrated the Transformer [25] architecture into our model. Originally designed for NLP, the original Transformer model comprises 2 primary modules: the encoder and the decoder. As depicted in Fig. 2, the encoder comprises 6 identical layers, with each layer containing 2 sublayers: a multihead self-attention layer and a fully connected feed-forward layer. Analogously, the decoder encompasses 6 layers, with each layer incorporating a third sublayer that performs multihead attention over the output of the encoder stack. Furthermore, both the encoder and the decoder apply a residual connection after each sublayer.

**Fig. 3.**
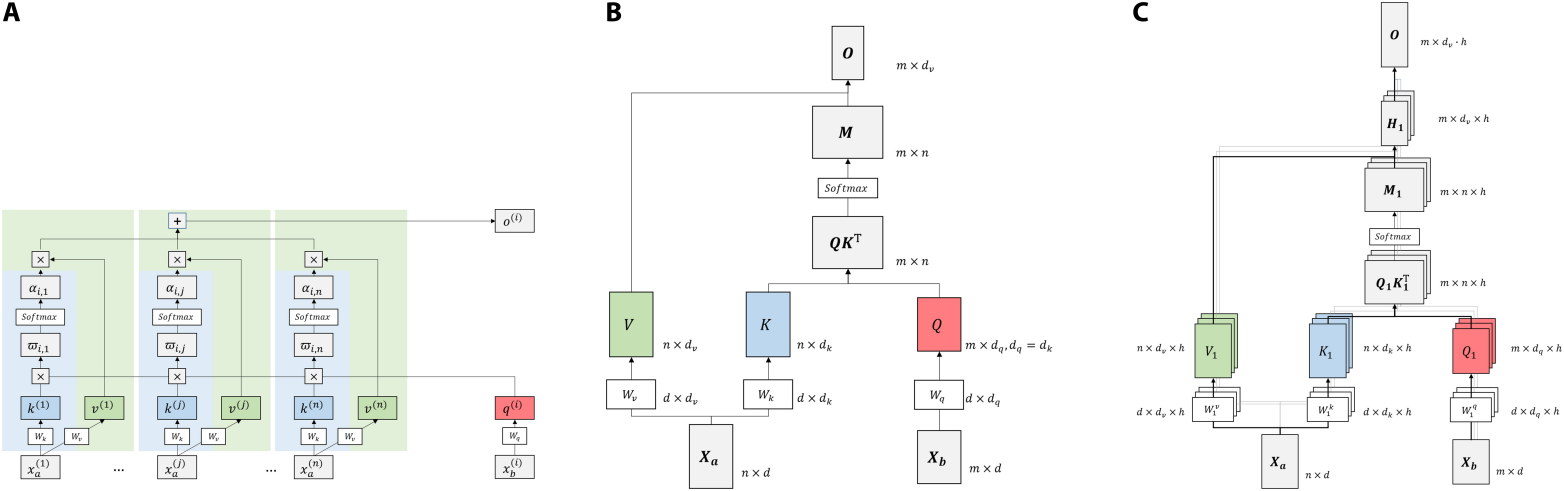
Attention mechanism. (A) Scaled dot-product attention for one step. (B) Calculation of scaled dot-product attention. (C) Calculation of multihead attention.

The Transformer mainly benefits from the attention mechanism, which maps a query and a set of key–value pairs to an output. The output is calculated as a weighted sum of the values, where the weights are determined by the query and associated keys. Specifically, the attention mechanism used in the Transformer is called “scaled dot-product attention”. As illustrated in Fig. 3A and B, the input of attention may involve 2 inputs $X_{a}\in R^{n\times d_{\text{model}}}$ (for encoder) and $X_{b}\in R^{m\times d_{\text{model}}}$ (for decoder), and the inputs are projected into queries and keys of dimension $d_{k}$ and values of dimension $d_{v}$. For ith step of the attention, the output is a weighted summary of values, and the weight $\alpha_{i,j}$ is culculated as follows:

$$\alpha_{i,j}=\text{softmax}\left(\frac{q^{\left(i\right)}k^{\left(j\right)T}}{\sqrt{d_{k}}}\right)$$ (5)

and the output of ith step is defiened as follows:

$$o_{i}=\sum_{j}\alpha_{i,j}\cdot v^{\left(j\right)}$$ (6)

Overall, the attention could be culculated on a set of queries simultaneously, packed together into a matrix $Q\in R^{m\times d_{q}}$. The keys and values are also packed together into matrices $K\in R^{n\times d_{k}}$ and $V\in R^{n\times d_{v}}$. When calculating self-attention, Q,K,andV are projected from the same input, and n=m. The attention is defined as follows:

$$\text{Attention}\left(Q,\;K,\;V\right)=\text{softmax}\left(\frac{QK^{T}}{\sqrt{d_{k}}}\right)V$$ (7)

Moreover, the Transformer utilized a mechanism to compute attention in parallel named multihead attention. As illustrated in Fig. 3C, in multihead attention, the queries, keys, and values are linearly projected h times (representing h heads). Subsequently, all h projections are concatenated and linearly projected again to yield the final output. The multihead attention is defined as follows:

$$\begin{array}{c} \text{MultiHead}\left(Q,\;K,\;V\right)=\text{Concat}\left({\text{head}}_{1},\ldots ,\;{\text{head}}_{h}\right)W^{O}\\ \text{where}\;{\text{head}}_{i}=\text{Attention}\left(Q_{i},\;K_{i},\;V_{i}\right) \end{array}$$ (8)

where the linear projections are weight matrices $W_{i}^{Q}\in R^{d_{\text{model}}\times d_{k}}$, $W_{i}^{K}\in R^{d_{\text{model}}\times d_{k}}$, $W_{i}^{V}\in R^{d_{\text{model}}\times d_{v}}$, and $W^{O}\in R^{h\cdot d_{v}\times d_{\text{model}}}$. Here, $d_{\text{model}}$ denotes the embedding dimension of the Transformer, and $d_{k}=d_{v}=\frac{d_{\text{model}}}{h}$.

### Background EEG mixing

The BGMix strategy is grounded in the neurophysiological understanding that EEG-BCI signals are composed of multiple components, each reflecting distinct brain activities [32]. Among these, certain components correspond to background neural processes that are not directly involved in the cognitive or motor tasks targeted in BCI applications. These task-irrelevant components can be regarded as background noise. In the broader field of data augmentation, a well-recognized approach involves the manipulation of background noise to increase data diversity and enhance model robustness [33]. Within the EEG domain, recent studies have demonstrated that component swapping—interchanging specific signal components between samples—can serve as an effective augmentation technique [34,35]. Drawing upon both neurophysiological principles and empirical engineering experience, we propose the BGMix strategy, which generates novel training samples by selectively swapping background components between EEG trials from different classes. This targeted manipulation preserves task-relevant information while introducing structured variability in the background activity, thereby improving model generalization and resilience to non-task-related fluctuations commonly observed in EEG recordings.

#### Task-related component and background EEG

**Fig. 4.**
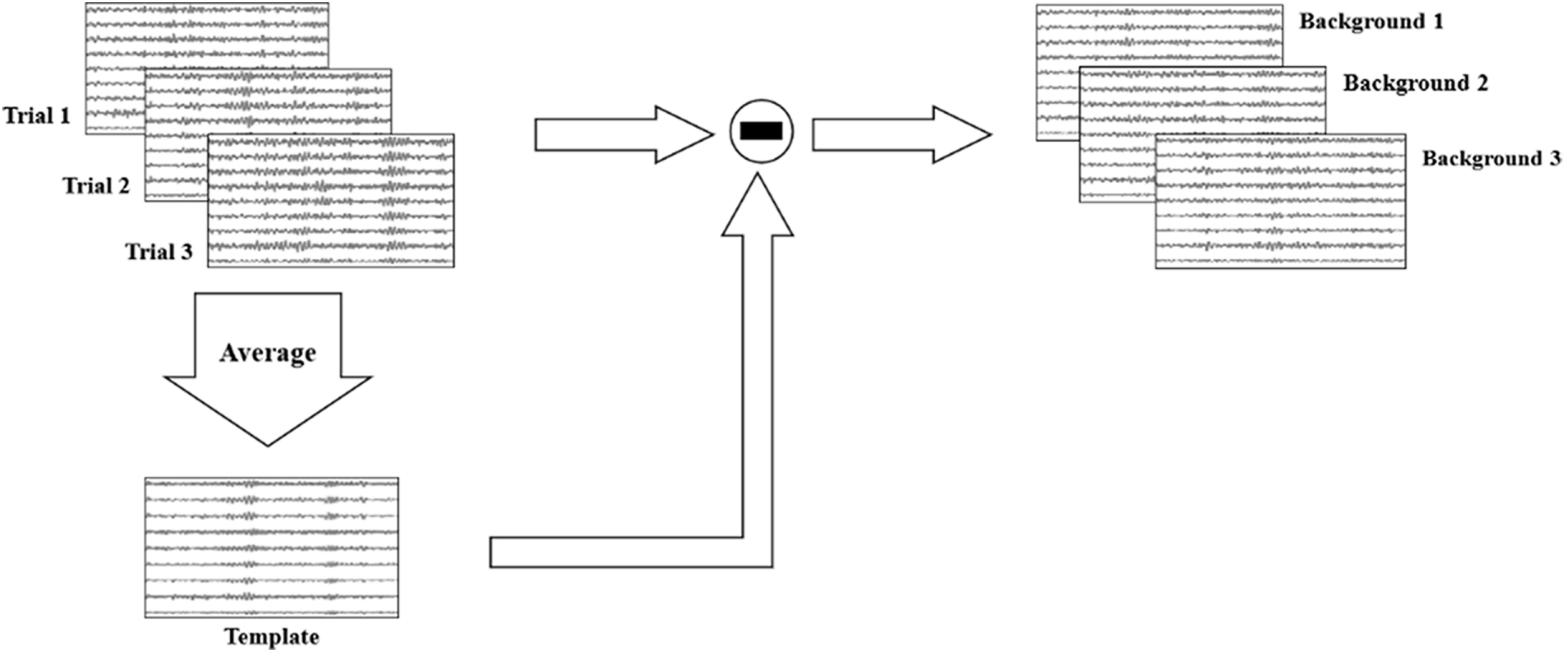
The process to obtain the average template and background EEG signals from EEG trials within the same class.

This section defines the task-related component and background EEG used in this study. The SSVEP signal is commonly conceptualized as a combination of 2 sources: the task-related component and the background EEG (task-unrelated component). While the distributions of task-related components remain consistent under the same stimulus, the background EEG signals vary significantly. When averaging *N* trials, the amplitude of the background EEG in the averaged template reduces to $1/\sqrt{n}$ of that in a single trial [36]. Consequently, in this study, the intertrial averaged template represents the task-related component, while the difference between the single-trial SSVEP and the template represents the background EEG (as depicted in Fig. 4).

Specifically, the single-trial SSVEP signal is denoted as $x_{h}^{m}\in R^{N_{c}\times N_{s}}$, $h\in 1,2,\ldots ,N_{t}$, and $m\in 1,2,\ldots ,N_{f}$, where *m* is the index of the stimulus and *h* is the index of the trial. $N_{t},N_{f},N_{c},\text{and}\;N_{s}$ are the number of trials, stimuli, channels, and sample points, respectively. Then, the averaged template is defined as follows:

$${\bar{x}}_{m}=\frac{1}{N_{t}}\sum_{h=1}^{N}x_{h}^{m}$$ (9)

and the background EEG is then defined as follows:

$$\mathrm{Bg}=x_{h}^{m}-{\bar{x}}_{m}$$ (10)

#### Mixing strategy

Denote $x\in R^{N_{c}\times N_{s}}$ as a sample of EEG data and y is the corresponding label. The purpose of the BGMix algorithm is to generate a new sample $\left(\tilde{x},\;\tilde{y}\right)$ from $\left(x,\;y\right)$ and $\left(x^{\prime },\;y^{\prime }\right)$, where $y\neq y^{\prime }$. BGMix is described as follows:

$$\tilde{x}=\text{Template}+{\mathrm{Bg}}^{\prime }$$ (11)

$$\tilde{y}=\lambda \cdot y_{i}+\left(1-\lambda \right)\cdot y_{j}$$ (12)

Here, for each subject, Template is the intertrial averaged template of x and $Bg^{\prime }$ is the background EEG of $x^{\prime }$. The fusion of the labels is performed when computing the loss of the deep learning in each training step, which is described as follows:

$$\begin{array}{c} \text{loss}=\lambda \cdot \text{loss}(y,\text{model}\left(\tilde{x}\right)+\\ \left(1-\lambda \right)\cdot \text{loss}(y^{\prime },\text{model}\left(\text{model}\left({\tilde{x}}^{\prime }\right)\right) \end{array}$$ (13)

Here, “model” denotes the deep learning model, and “loss” denotes the loss function. For each training step, λ is chosen from the Beta distribution.

**Fig. 5.**
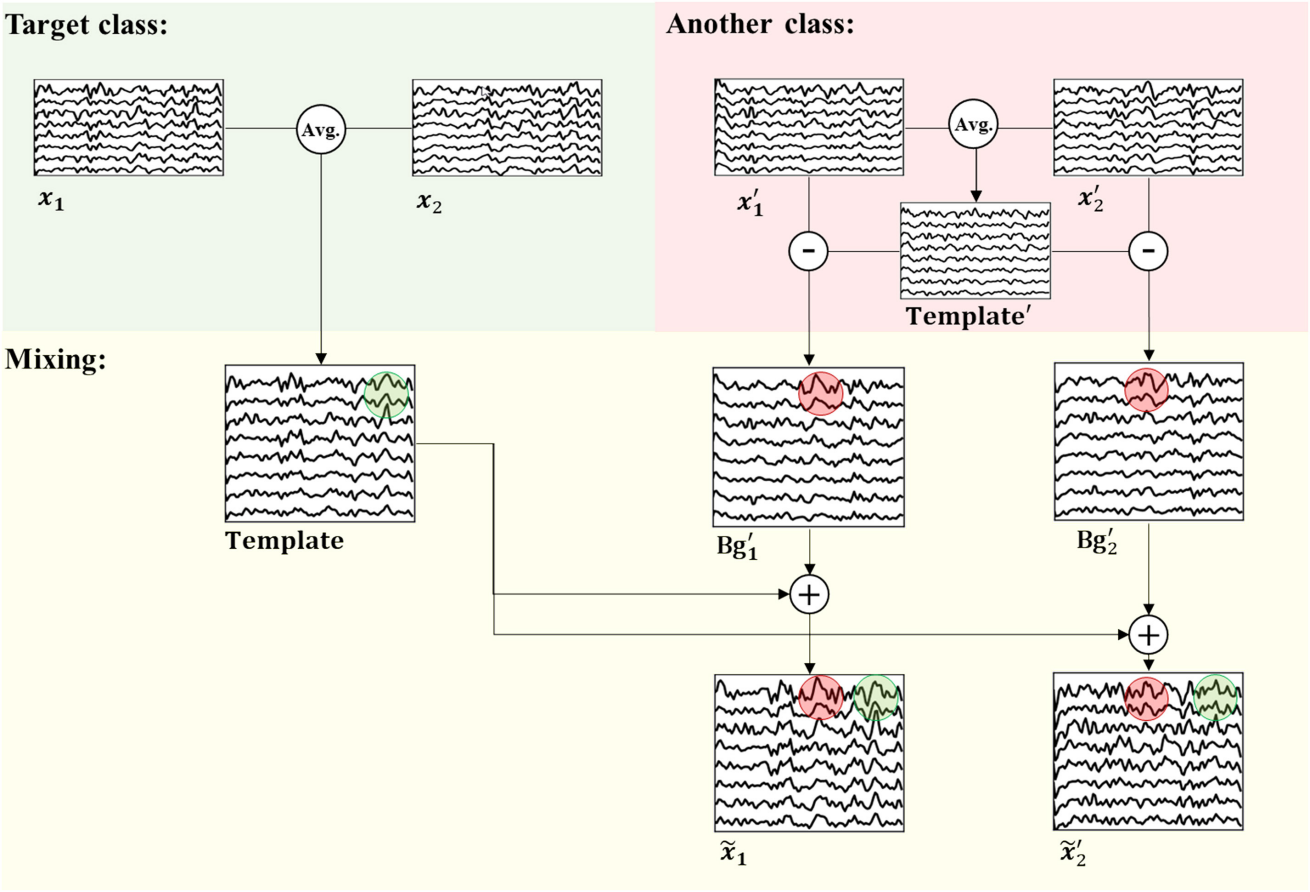
Demonstration of generating samples from raw SSVEP samples, average template, and background EEG signals. The intuitive SSVEP feature (green part) from the template and the disturbance from background EEG (brown part) are reserved in the generated samples.

Fig. 5 illustrates the process of generating new samples from 2 distinct classes using the BGMix strategy. For a target class, we randomly select $n\left(2\leq n\leq N_{t}\right)$ trials of training samples $\left[x_{1},\;x_{2},\ldots ,\;x_{n}\right]$ to build the template signal (intuitive feature is highlighted as a green circle), with Fig. 5 using *n* = 2 as an example. To create the background EEG $\left[{\mathrm{Bg}}_{1}^{\prime },\;{\mathrm{Bg}}_{2}^{\prime },\ldots ,\;{\mathrm{Bg}}_{n}^{\prime }\right]$, we randomly select *n* trials of samples $\left[x_{1}^{\prime },\;x_{2}^{\prime },\ldots ,\;x_{n}^{\prime }\right]$ from another class, construct the template signal, and subtract the template from the selected trials. By combining the template signal with the background EEG signals (intuitive features are highlighted as red circles) from different classes, we generate *n* new training samples. Algorithm S1 provides the Python code of the BGMix strategy.

### Augment EEG Transformer

**Fig. 6.**
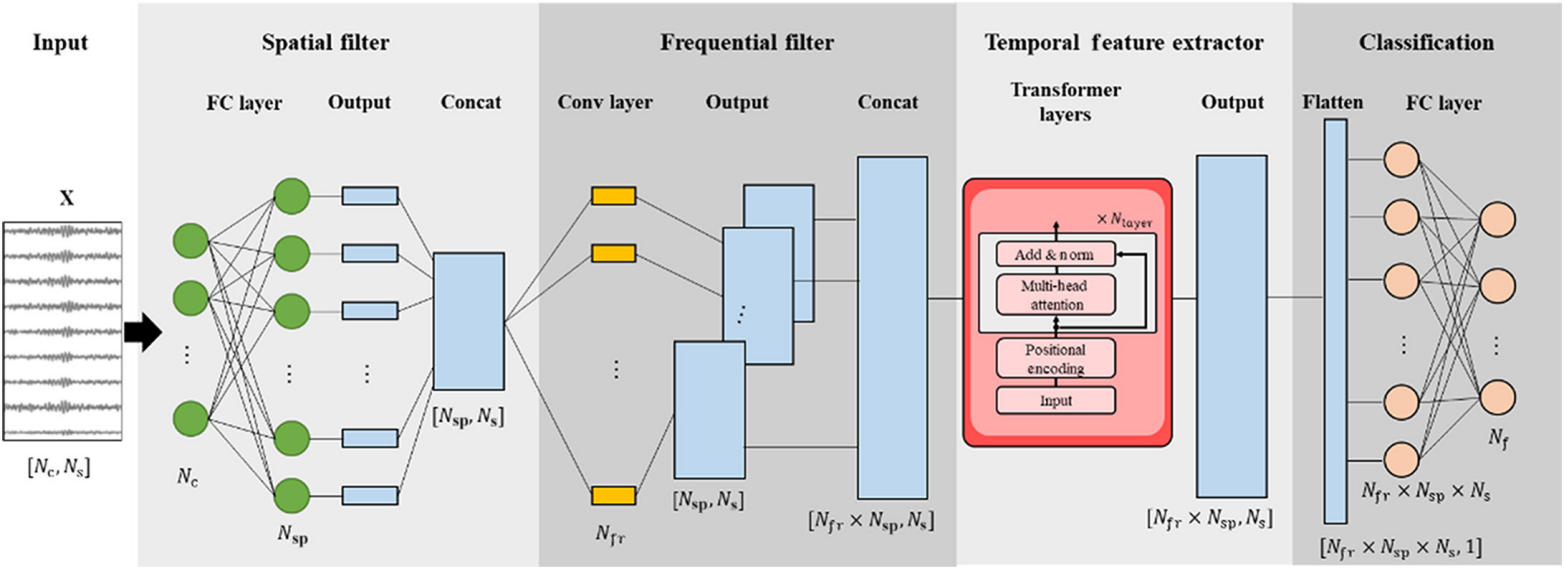
The overall structure of the TF model. The modules from left to right are a fully connected layer acting as spatial filters, a convolutional layer functioning as frequential filters, and a Transformer encoder module serving as the temporal feature extractor. A classification layer follows the AETF to output the prediction.

In this section, we propose a TF model to decode SSVEP signals. Combined with the BGMix strategy, we name this model the AETF. Fig. 6 illustrates the structure of the TF model, and Table 1 provides details. The model comprises 3 modules: the first module is a fully connected layer serving as the spatial filters, the second module is a convolutional layer acting as frequential filters, and the final module features a multilayer Transformer encoder designed to extract the temporal information from the sequence. Since no data compression module like pooling layers exists, the model preserves the entire sequence information and embeds it effectively. Moreover, the transformer layers with attention mechanisms enable the model to better extract the sequence information by focusing on critical features.

**Table 1.** Architecture of AETF model

Layer	Input	Output	Kernel	Activation	Padding
FC	$\left(1,\;N_{c},\;N_{s}\right)$	$\left(1,\;N_{\mathrm{sp}},\;N_{s}\right)$		Tanh	
Conv2D	$\left(1,\;N_{c},\;N_{s}\right)$	$\left(N_{\mathrm{fr}},\;N_{\mathrm{sp}},\;N_{s}\right)$	$\left(1,\;\text{sample}\_\text{rate}/2\right)$	Elu	Same
BatchNorm2D	$\left(N_{\mathrm{fr}},\;N_{\mathrm{sp}},\;N_{s}\right)$	$\left(N_{\mathrm{fr}},\;N_{\mathrm{sp}},\;N_{s}\right)$			
PE	$\left(N_{\mathrm{fr}}\times N_{\mathrm{sp}},\;N_{s}\right)$	$\left(N_{\mathrm{fr}}\times N_{\mathrm{sp}},\;N_{s}\right)$			
TF Encoder	$\left(N_{\mathrm{fr}}\times N_{\mathrm{sp}},\;N_{s}\right)$	$\left(N_{\mathrm{fr}}\times N_{\mathrm{sp}},\;N_{s}\right)$		Leaky Relu	
FC (classification layer)	$\left(N_{\mathrm{fr}}\times N_{\mathrm{sp}}\times N_{s}\right)$	$N_{f}$			

#### Fully connected layer

The SSVEP sample is first fed into a fully connected layer, which serves as spatial filters. We denote the input SSVEP sample as $x\in R^{N_{c}\times N_{s}}$, where $N_{c}$ is the number of electrodes and $N_{s}$ is the number of sample points, and the number of classes is denoted as $N_{f}$. The input dimension of the fully connected layer is $N_{c}$, and the output dimension is $N_{\mathrm{sp}}$, which is the number of spatial filters. The activation function used is tanh. Each cell in the output layer represents a spatial filter, forming a nonlinear combination of all electrodes. The output of the fully connected layer is denoted as $O_{\mathrm{FC}}\in R^{N_{\mathrm{sp}}\times N_{s}}$.

#### Convolutional layer

Continuing from the fully connected layer, a convolutional layer is applied to extract features in the frequency domain. This module draws inspiration from the EEGNet, with the kernel size set to $\left(1,\;\text{sample}\_\text{rate}/2\right)$. As noted in the EEGNet study, setting the kernel length to half the sampling rate enables the model to capture frequency information at 2 Hz and above. The input to the convolutional layer is represented as a one-channel 2-dimensional (2D) vector ($O_{\mathrm{FC}}\in R^{N_{\mathrm{sp}}\times N_{s}}$), while the output channel equals the number of frequential filters $N_{f}r$. Zero-padding is applied to maintain the output size equivalent to the input size, resulting in an output of $O_{\text{Conv}}\in R^{N_{\mathrm{fr}}\times N_{\mathrm{sp}}\times N_{s}}$. To fit the following transformer layers, the output is flattened along the time domain to be the size of $\left(N_{\mathrm{fr}}\times N_{\mathrm{sp}},\;N_{s}\right)$.

#### Positional encoding

Positional encoding incorporates positional information before passing the data to the transformer layers. The positional encodings share the same dimension as the output of the convolutional layer $\left(N_{\mathrm{fr}}\times N_{\mathrm{sp}}\right)$, allowing the 2 to be summed. Here, we use sine and cosine functions of different frequencies as the positional encoding function:

$$\mathrm{PE}\left(\mathrm{pos},\;2i\right)=\text{sin}\left(\frac{\mathrm{pos}}{10,000^{2i/d_{\text{model}}}}\right)$$ (14)

$$\begin{array}{c} \mathrm{PE}\left(\mathrm{pos},\;2i+1\right)=\text{cos}\left(\frac{\mathrm{pos}}{10,000^{2i/d_{\text{model}}}}\right) \end{array}$$ (15)

where pos is the position and i is the index of the input dimension. For any position k, $\mathrm{PE}\left(k\right)$ produces a unique position vector for the network, enabling the model to effectively learn position information.

#### Transformer layers

The Transformer module comprises multiple transformer encoder layers, with the number of layers denoted as $N_{\text{layer}}$. Each transformer layer incorporates a multihead self-attention layer, followed by a position-wise fully connected layer. A residual connection is established between the input and the fully connected layer. The input dimension matches the output of the convolutional layer $\left(N_{\mathrm{fr}}\times N_{\mathrm{sp}}\right)$, and we denote the embedding dimension as $N_{\mathrm{tf}}$. The number of heads is configured to match the number of the frequential filters $N_{\mathrm{fr}}$, mimicking the operation of filter banks. The output dimension of each transformer layer is consistent with the input dimension.

#### Classification layer

For the classification task, we deployed a fully connected layer to predict the class of the input sample, as shown in Fig. 6. The output of the AETF is flattened to the size of $\left(N_{\mathrm{fr}}\times N_{\mathrm{sp}}\times N_{s},\;1\right)$, and the input and output dimensions of the fully connected layer are$N_{\mathrm{fr}}\times N_{\mathrm{sp}}\times N_{s}$ and $N_{f}$.

### Datasets

**Fig. 7.**
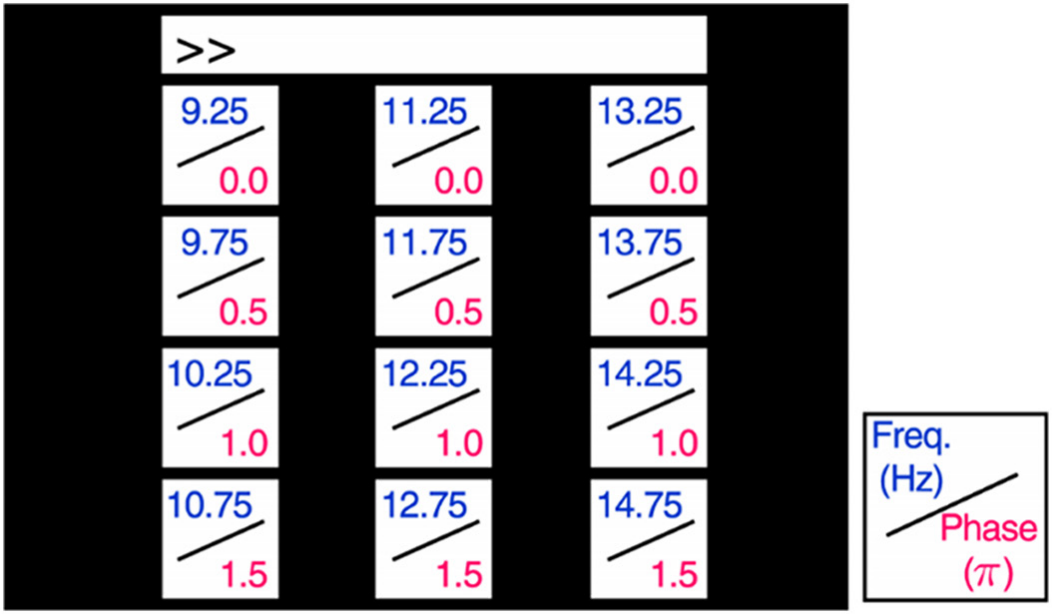
Stimulation parameters of dataset I.

This study employs 2 publicly available SSVEP datasets, referred to as dataset I and dataset II, to evaluate the effectiveness of the AETF. In dataset I [37], 10 subjects participated in an SSVEP-BCI experiment, during which each subject completed 15 blocks of experimental tasks. Each block comprised 12 trials corresponding to 12 stimuli, with each stimulus flickering for 4 s. Fig. 7 illustrates the stimulus configuration. The EEG signals from each subject, recorded via 8 channels, were sampled at a rate of 256 Hz.

**Fig. 8.**
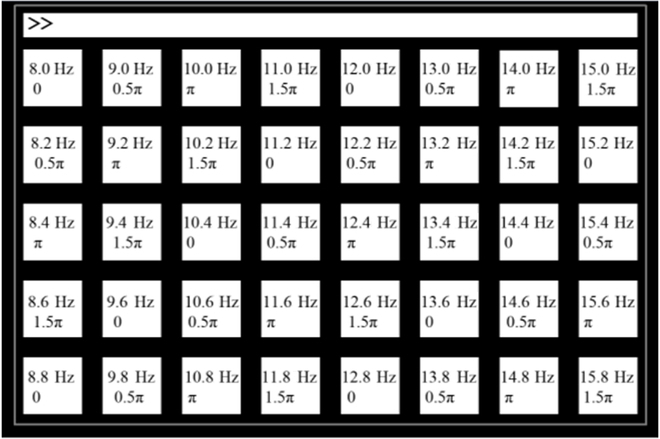
Stimulation parameters of dataset II.

In dataset II [38], 64-channel EEG signals from 35 subjects were recorded during an offline SSVEP-BCI experiment. Each subject’s data included 6 blocks, with each block comprising 40 trials. During each trial, 40 character stimuli flickered for 5 s. Fig. 8 illustrates the stimulus configuration. We recorded the EEG signals at a sampling rate of 1,000 Hz and then downsampled them to 250 Hz.

For evaluation purposes, this study used all 8 channels from dataset I (PO3, PO4, PO7, PO8, POz, O1, O2, and OZ) and 9 channels from dataset II (Pz, PO3, PO4, PO5, PO6, POz, O1, O2, and Oz). The signals from both datasets were band-pass filtered from 8 to 90 Hz using a Chebyshev filter. See the Data Availability section for dataset access.

### Experiment settings

#### Training details

In this study, the model parameter settings remain consistent across both datasets. The number of spatial filters ($N_{\mathrm{sp}}$) is set to be twice the number of electrodes of the input SSVEP signal (i.e., 16 for dataset I and 18 for dataset II), while the number of frequential filters ($N_{\mathrm{fr}}$) is set to 8. A 2-layer transformer encoder ($N_{\text{layer}}=2$) functions as the temporal feature extraction module of the model. The embedding dimension of the transformer layer ($N_{\mathrm{tf}}$) is set to be $N_{\mathrm{sp}}\times N_{\mathrm{fr}}$. Details of parameter settings could be found in Table 1. We trained all the deep learning models using the Adam optimizer with a certain weight decay to avoid overfitting. The experiments are performed on a deep learning workstation with an AMD Ryzen Threadripper 1900X and an NVIDIA RTX 2080 TI. We developed the model using the PyTorch library; MetaBCI (https://github.com/TBC-TJU/MetaBCI) facilitated signal preprocessing and subsequent deployment of the baseline model. See Data Availability for further information.

This study adopts a series of training techniques aimed at enhancing model generalization across subjects. The first technique involves a 2-stage training procedure, which can be viewed as a simplified form of transfer learning designed to mitigate domain shifts arising from intersubject variability. In the first training stage, an intersubject model is trained using data pooled from all subjects in the dataset. This step serves to align cross-subject variability and encode common, stable SSVEP-related features into the model. In the second training stage, a subject-specific model is fine-tuned for each individual subject. These models are initialized with the weights of the intersubject model and subsequently adapted using the training samples from the corresponding subject, thereby tailoring the model to subject-specific characteristics while leveraging shared representations learned during the first stage.

To evaluate model performance, we adopt *K*-fold cross-validation, where the data for each subject are partitioned at the trial level. In each fold, we select one trial per class from each subject to form the test set, and the remaining trials are used to form the training and validation sets for each subject. Typically, one additional trial from the remaining data is designated as the validation set, and the rest are used for training. When applying the BGMix strategy for data augmentation, the augmented samples generated from both the original training and validation sets are combined to form a new expanded training set. Meanwhile, the original (non-augmented) training and validation sets are concatenated to constitute the new validation set. Notably, the BGMix strategy is only conducted in the second training stage to generate subject-specific training samples.

Throughout the training process, loss values are calculated for both the training and validation sets, and model parameters are saved when the validation loss reaches its minimum.

Additionally, an early-stopping strategy is applied to mitigate overfitting, terminating the training process if the validation loss does not decrease over several steps.

In this study, we ensured the diversity of the generated samples by varying the number of trials used to construct both the template signals and background EEG components within each subject. This strategy was designed to introduce rich variability in the synthesized SSVEP signals while preserving the underlying subject-specific characteristics. Specifically, for dataset I, we augmented the original SSVEP samples by a factor of 40 using 2 trials to form the template and background noise, and by an additional factor of 40 using 3 trials, resulting in an overall augmentation of 80 times the original sample size. For dataset II, where the number of available training trials per subject was more limited, we applied 3 configurations using 2, 3, and 4 trials, respectively, each yielding an augmentation of 40 times. This resulted in a total expansion of 120 times the original number of samples.

The augmentation scale was selected based on an empirical trade-off between model performance and computational cost, which is further analyzed in the discussion section to justify its effectiveness and efficiency.

#### Baseline methods

##### Augmentation strategies

To evaluate the effectiveness of BGMix, we compared its performance with several classical EEG data augmentation strategies. These baseline methods fall into 3 categories, encompassing 6 specific techniques: (a) temporal domain augmentation, including Gaussian noise [39], time reverse [40], and smooth time masking [41]; (b) frequency domain augmentation, including frequency shift [40] and Fourier transform surrogate [42]; and (c) spatial domain augmentation, represented by channel shuffling [43]. All 6 methods were implemented using the Braindecode (http://github.com/braindecode/braindecode) library, which is built on the PyTorch framework. Additionally, a simple Mixup strategy is also used to compare its performance between the BGMix strategy.

##### Deep learning models

In this study, we used 3 widely recognized deep learning models applied in the field of EEG analysis: ShallowConvNet, DeepConvNet [11], and EEGNet [12]. ShallowConvNet features a simple architecture that includes a temporal convolution layer with a large kernel size, followed by a spatial filter, squaring nonlinearity, mean pooling, and a logarithmic activation function, all optimized jointly for decoding band power features from raw EEG signals. DeepConvNet employs a more complex structure, which is characterized by its multilayer architecture, including 5 convolutional layers designed to extract increasingly complex features from raw EEG data. It employs advanced techniques such as batch normalization and dropout to improve training efficiency and performance. EEGNet is a compact CNN specifically tailored for EEG classification, comprising 3 convolutional layers. The first layer is a temporal convolutional layer aimed at learning frequency features, followed by a depthwise convolutional layer for capturing spatial features, and a separable convolutional layer to merge temporal features from various feature maps. In this study, EEGNet is adapted according to the specifications outlined in [44] to suit SSVEP signals. The implementations of these models also follows guidelines of the Braindecode library. See Data Availability for more details of model parameter settings.

##### Task-related component analysis

Task-related component analysis (TRCA) [45] utilizes subject-specific templates to extract the task-related components from the test data. Specifically, TRCA considers the multichannel EEG signal $x\left(t\right)$ as being composed of a linear combination of task-related component $s\left(t\right)$ and task-unrelated signal $n\left(t\right)$:

$$x_{j}\left(t\right)=a_{1,j}\cdot s\left(t\right)+a_{2,j}\cdot n\left(t\right),j=1,2,\ldots ,N_{c}$$ (16)

where j is the index of the channel, $N_{c}$ is the number of channels, and $a_{1,j}$ and $a_{2,j}$ are mixing weights for task-related components. Thus, the task-related component could be recovered as follows:

$$y\left(t\right)=\sum_{j=1}^{N_{c}}w_{j}x_{j}=\sum_{j=1}^{N_{c}}w_{j}\left(a_{1,j}\cdot s\left(t\right)+a_{2,j}\cdot n\left(t\right)\right)$$ (17)

Ideally, the problem has a solution of $y\left(t\right)=s\left(t\right)$, which can be solved by maximizing intertrial covariance:

$$w^{T}Sw=\sum_{h_{1},h_{2}=1}^{N_{t}}\sum_{h_{1}\neq h_{2}}^{N_{c}}w_{j_{1}}w_{j_{2}}\mathrm{Cov}\left(x_{j_{1}}^{h_{1}}\left(t\right),\;x_{j_{2}}^{h_{2}}\left(t\right)\right)$$ (18)

To obtain a finite solution, a constraint of variance is added:

$$\mathrm{Var}\left(y\left(t\right)\right)=\sum_{j_{1},j_{2}=1}^{N_{\mathrm{ch}}}w_{j_{1}}w_{j_{2}}\mathrm{Cov}\left(x_{j_{1}}\left(t\right),\;x_{j_{2}}\left(t\right)\right)=w^{T}Qw$$ (19)

Then, the spatial filter of the TRCA could be solved as follows:

$$\hat{w}=\text{arg}\;\underset{w}{\text{max}}\frac{w^{T}Sw}{w^{T}Qw}$$ (20)

By applying the filter bank method, the ensemble TRCA (eTRCA) is proposed to improve the SSVEP decoding efficiency. In our study, the lower and upper cutoff frequencies of the *m*th sub-band filter were set to *m* × 8 Hz and 90 Hz, and the number of filters is set to 3. The eTRCA employs an ensemble spatial filter:

$$W=\left[w_{1},\;w_{2},\ldots ,\;w_{N_{f}}\right]\in R^{N_{c}\times N_{f}}$$ (21)

where $N_{f}$ denotes the number of the classes. See more details in Ref. [45].

##### Task discriminant component analysis

The task discriminant component analysis (TDCA) [46] reduces the redundancy in the spatial filters of TRCA and increases the effectiveness of SSVEP decoding. For a training trial of EEG data $X\in R^{N_{c}\times N_{s}}$, TDCA fist generated the augmented data as follows:

$$\tilde{X}={\left[X^{T},\;X_{1}^{T},\ldots ,\;X_{l}^{T}\right]}^{T}$$ (22)

where $\tilde{X}\in R^{\left(l+1\right)N_{c}\times N_{s}}$ is the augmented EEG trial and $X_{l}\in R^{N_{c}\times N_{s}}$ denotes the EEG signal with l points delay. For test trials, the augmented EEG is padded with zeros if the delayed points exceed the data length. Then, $\tilde{X}$ is projected onto the subspace spanned by the reference signal Y:

$${\tilde{X}}_{p}=\tilde{X}P_{i}$$ (23)

$$P_{i}=QQ^{T}$$ (24)

$$Y_{i}=\left[\begin{array}{c} \text{sin}\left(2\pi f_{i}t^{T}\right)\\ \text{cos}\left(2\pi f_{i}t^{T}\right)\\ \ldots \\ \text{sin}\left(2\pi N_{h}\;f_{i}t^{T}\right)\\ \text{cos}\left(2\pi N_{h}\;f_{i}t^{T}\right) \end{array}\right]=\mathrm{QR}$$ (25)

Here, $Y_{i}$ is the reference signal of *i*th class and *Q* derives from the orthogonal-triangular (QR) decomposition of $Y_{i}$. $N_{h}$ is the number of harmonics, $f_{i}$ is the frequency of *i*th stimulus, and $f_{s}$ is the sampling rate. $t=\left[1/f_{s},...,N_{s}/f_{s}\right]$. Next, a secondary augmented EEG trial is constructed as follows:

$$X_{a}=\left[\tilde{X},\;{\tilde{X}}_{p}\right]$$ (26)

For *i*th class, the class center is defined as follows:

$${\bar{X}}_{a}^{a}=\frac{1}{N_{t}}\sum_{i=1}^{N_{t}}X_{a}^{\left(i\right)}$$ (27)

Finally, to find projection directions to separate trials from all classes, a 2D linear discriminant analysis (LDA) is conducted, which uses Fisher criterion to derive the projection directions:

$$\underset{w}{\text{max}}\frac{\mathrm{tr}\left(W^{T}S_{b}W\right)}{r\left(W^{T}S_{w}W\right)}$$ (28)

where $S_{b}$ is the between-class scatter matrix and $S_{w}$ is the within-class scatter matrix. In TDCA, the scatter matrices could be obtained by:

$$S_{b}=H_{b}H_{b}^{T}$$ (29)

$$S_{w}=H_{w}H_{w}^{T}$$ (30)

Here, $H_{b}\in R^{\left(l+1\right)N_{c}\times 2N_{f}N_{s}}$ denotes between-class difference matrix, $H_{w}\in R^{\left(l+1\right)N_{c}\times 2N_{t}N_{s}}$ denotes within-class difference matrix, and they are defined as follows:

$$H_{b}=\frac{1}{\sqrt{N_{f}}}\left[{\bar{X}}_{a}^{1}-{\bar{X}}_{a}^{a},\ldots ,\;{\bar{X}}_{a}^{N_{c}}-{\bar{X}}_{a}^{a}\right]$$ (31)

$$\begin{array}{c} H_{w}=\frac{1}{\sqrt{N_{f}}}\left[X_{a}^{\left(1\right)}-\;{\bar{X}}_{a}^{1},\ldots ,X_{a}^{\left(N_{t}\right)}-{\bar{X}}_{a}^{\left(N_{t}\right)}\right] \end{array}$$ (32)

where ${\bar{X}}^{i}$ and $X^{\left(i\right)}$ refer to the 2D class centers of the *i*th class and the *i*th sample, respectively. In the following experiment, we set *N_h_* = 5 and *l* = 5.

#### Performance metric

##### Balanced accuracy

The balanced accuracy (BA) is used to evaluate the performance of the models in the SSVEP classification task. The BA could address the bias in the accuracy caused by the imbalance of samples from different categories. If there are *K* classes in total, the BA is defined as follows:

$$\mathrm{BA}=\frac{1}{K}\sum_{k=1}^{K}\frac{{\hat{N}}_{k}}{N_{k}}$$ (33)

where $N_{k}$ is the total number of observations in the *k*th class and ${\hat{N}}_{k}$ is the number of observations correctly identified as the same class label.

##### Information transfer rate

ITR is often used to measure the classification efficiency of BCI systems, which is defined as follows:

$$\begin{array}{c} \mathrm{ITR}=\frac{60}{T}\left[\log_{2}N+\text{P}{\text{log}}_{2}P+\left(1-P\right){\text{log}}_{2}\frac{\left(1-P\right)}{\left(N-1\right)}\right] \end{array}$$ (34)

where *N* is the number of stimuli, *P* is the accuracy, and *T* is the time consumed for each calculation process.

## Results

### Performance improvement with BGMix strategy

**Fig. 9.**
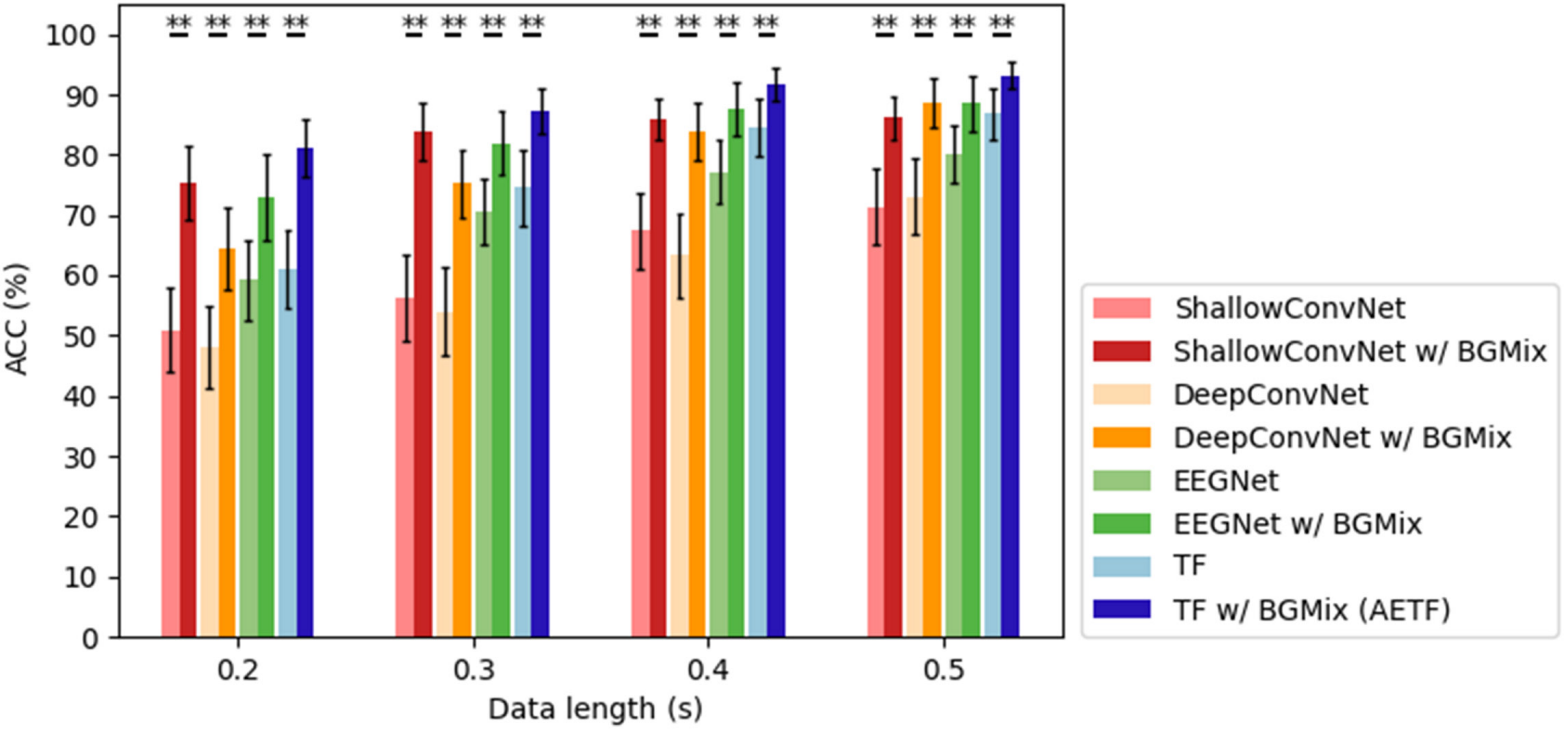
Accuracy comparison between w/ and w/o BGMix in dataset I. The asterisks indicate the significance of the improvement with BGMix (*P < 0.05, **P < 0.01, ***P < 0.001). The error bars represent standard error of the mean (SEM).

To evaluate the efficacy of BGMix, we compared the classification performance of deep learning models with and without the BGMix strategy. Specifically, we computed the average classification accuracy across 4 models (ShallowConvNet, DeepConvNet, EEGNet, and TF model) using varying training data lengths. For dataset I, we conducted Wilcoxon signed-rank tests to statistically compare the classification performance across each scenario. As depicted in Fig. 9, regardless of the model or training data length, the BGMix strategy resulted in a significant (*P* < 0.01) improvement in classification accuracy. The average accuracies of the 4 models rose by 21.39%, 18.44%, 11.06%, and 11.62%, respectively. Notably, the improvement became more pronounced with shorter training lengths. For instance, with 0.2 s of training data, the detailed subject-specific accuracy improvements appear in the Supplementary Materials. These results demonstrate the robustness of the BGMix strategy across subjects, as most subjects exhibited improved accuracies with both deep learning models.

**Fig. 10.**
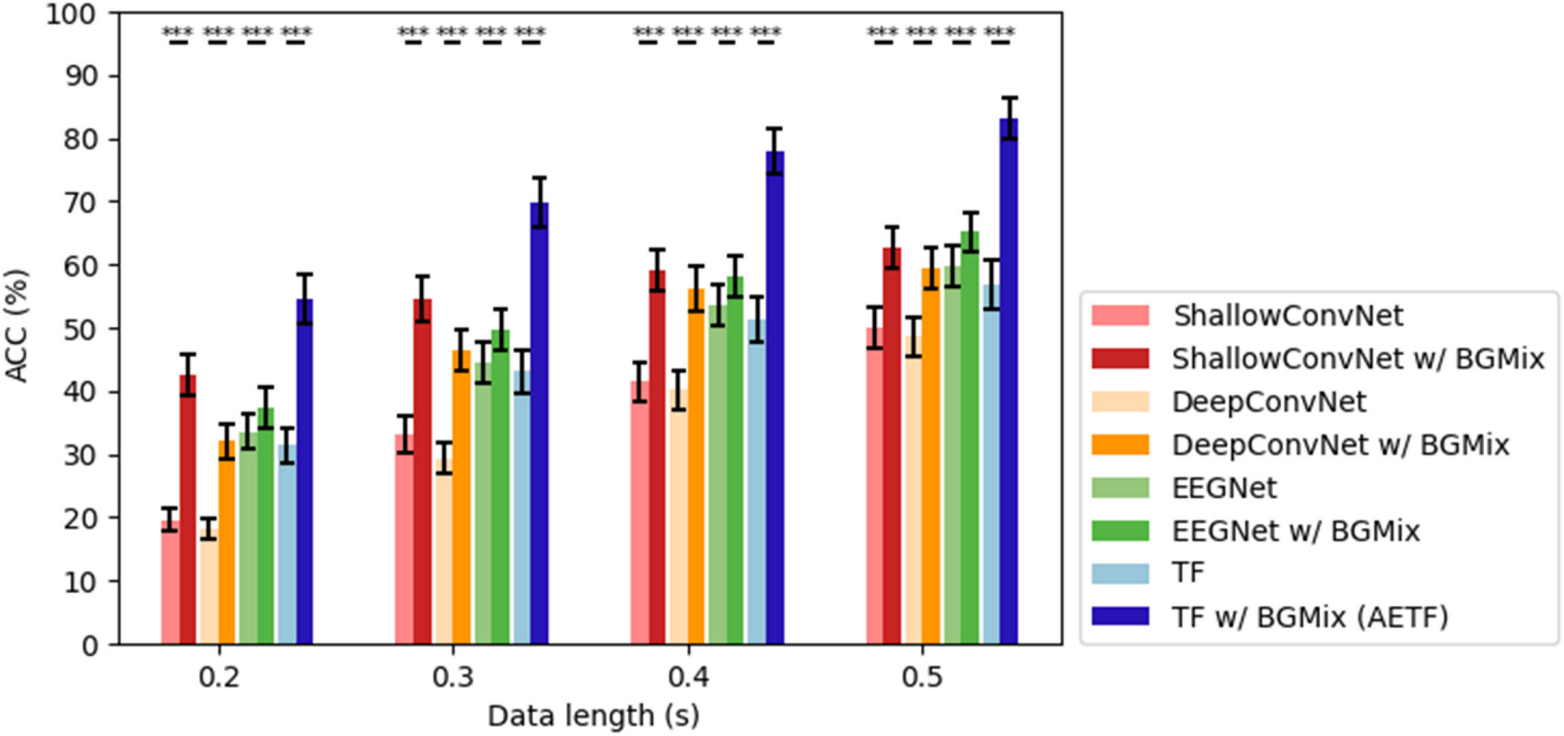
Accuracy comparison between w/ and w/o BGMix in dataset II. The asterisks indicate the significance of the improvement w/ BGMix (*P < 0.05, **P < 0.01, ***P < 0.001). The error bars represent SEM.

Fig. 10 shows the average classification accuracies obtained in dataset II. For each training data length, we conducted a paired *t* test to statistically compare the classification performance with and without the BGMix strategy. The average accuracies of the 4 models increased by 18.64%, 14.47%, 4.81%, and 25.17%, respectively. To validate the cross-subject robustness of BGMix, subject-specific classification performance appears in the appendix. The results indicate that, with the BGMix strategy, accuracies significantly improve irrespective of the training data length, and this improvement remains robust across subjects in dataset II. Additionally, unlike dataset I, the classification accuracy of TF model falls below the EEGNet. However, with the help of BGMix strategy, the AETF model achieved a significant increase, surpassing the accuracy of EEGNet with BGMix. This phenomenon might result from the limit number of intra-class trials, and the Transformer module requires sufficient data diversity to better extract high-dimension temporal features, making it perfectly suited to the BGMix strategy.

**Fig. 11.**
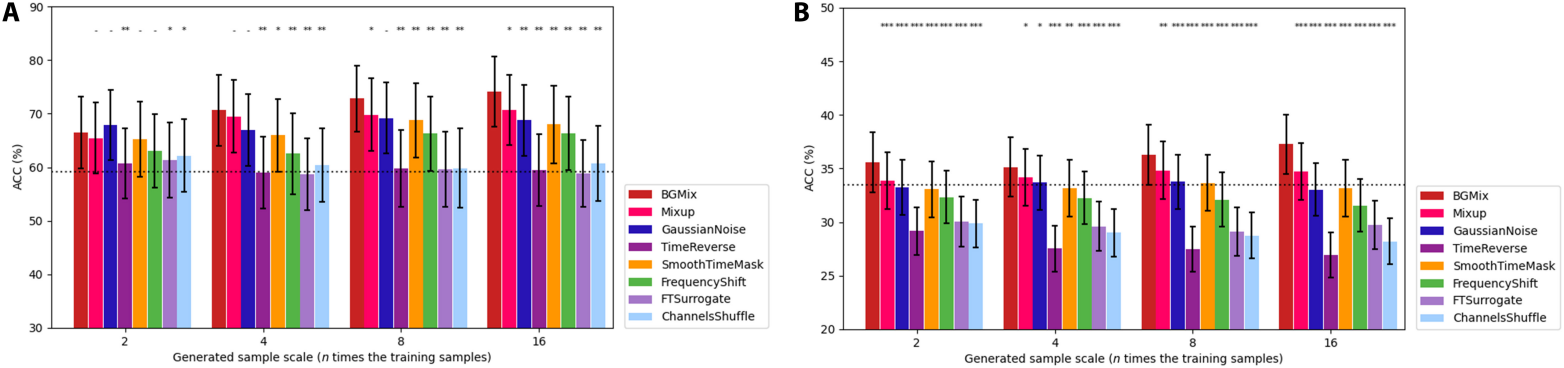
Accuracy comparison between the BGMix and other augmentation methods. Each of the baseline methods is compared with the BGMix strategy, and the black dot line indicates the model performance without data augmentation techniques. (A) Dataset I. (B) Dataset II. *P < 0.05, **P < 0.01, ***P < 0.001. The error bars represent SEM.

We then evaluated the classification performance of the BGMix strategy in comparison with baseline data augmentation methods, as illustrated in Fig. 11. As previously mentioned, for both datasets, 7 baseline methods, along with BGMix, were selected and analyzed under varying amounts of generated data. Specifically, the experiment was conducted using the EEGNet model with 0.2-s training samples, keeping all settings unchanged except for the data augmentation method. Under the same conditions, statistical tests were performed to compare BGMix with each baseline method. For dataset I, Wilcoxon signed-rank tests were used, while paired *t* tests were applied for dataset II, which serves as the default statistical approach in the subsequent analysis

The results indicate that BGMix achieved significantly superior performance in most cases and demonstrated improved effectiveness as the number of generated samples increased. In contrast, the performance of baseline methods did not improve with an increasing number of generated samples and, in some cases, even declined. This result highlights the limitations of conventional data augmentation techniques in generating sufficient high-quality training data. Consequently, the comparison experiment was concluded after generating 16 times the original training samples. However, our observations suggest that BGMix is capable of generating more diverse training samples. As mentioned earlier, the number of generated samples was set to 80 times the original dataset size for dataset I and 120 times for dataset II. These findings further validate the effectiveness of BGMix and underscore its potential to introduce greater diversity into training samples compared to conventional data augmentation methods.

### Ablation experiment of transformer module

**Fig. 12.**
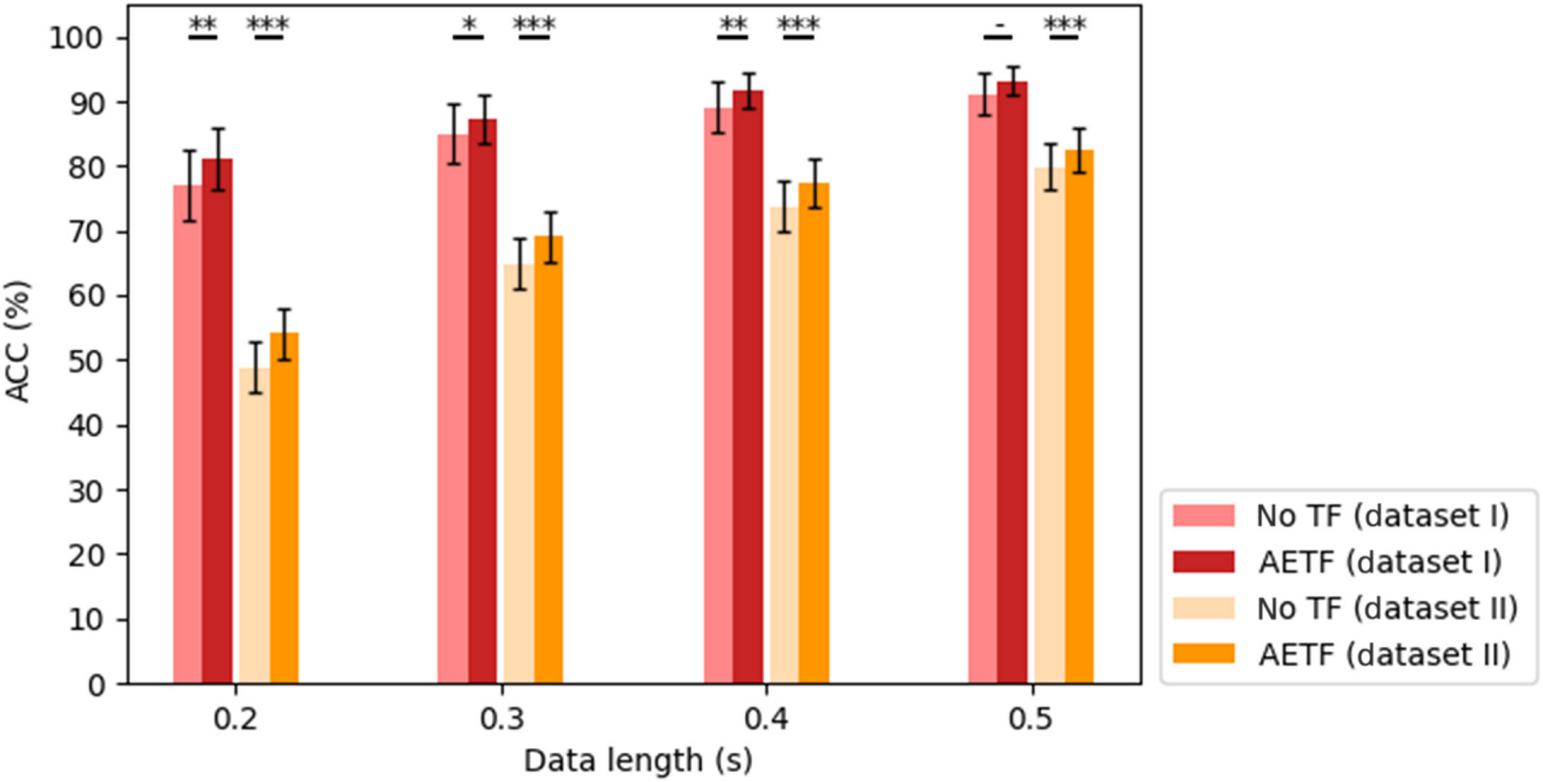
Ablation experiment. The classification performance of AETF with/without the Transformer module. The asterisks indicate significant improvement w/ the TF module (*P < 0.05, **P < 0.01, ***P < 0.001). The error bars represent SEM.

In this section, we conducted an ablation experiment on the AETF model to evaluate the effect of the Transformer module. As demonstrated in Fig. 12, removing the 2-layer Transformer module reduces the classification accuracy of AETF decreases, regardless of the training data length or the dataset. The average accuracy drops by 2.79% and 2.96% for each dataset, respectively. Moreover, the outstanding performance under short training data lengths of AETF declines significantly without the Transformer module, highlighting its importance in building a high-speed BCI decoding algorithms.

### Outstanding performance of AETF under short training data length

**Fig. 13.**
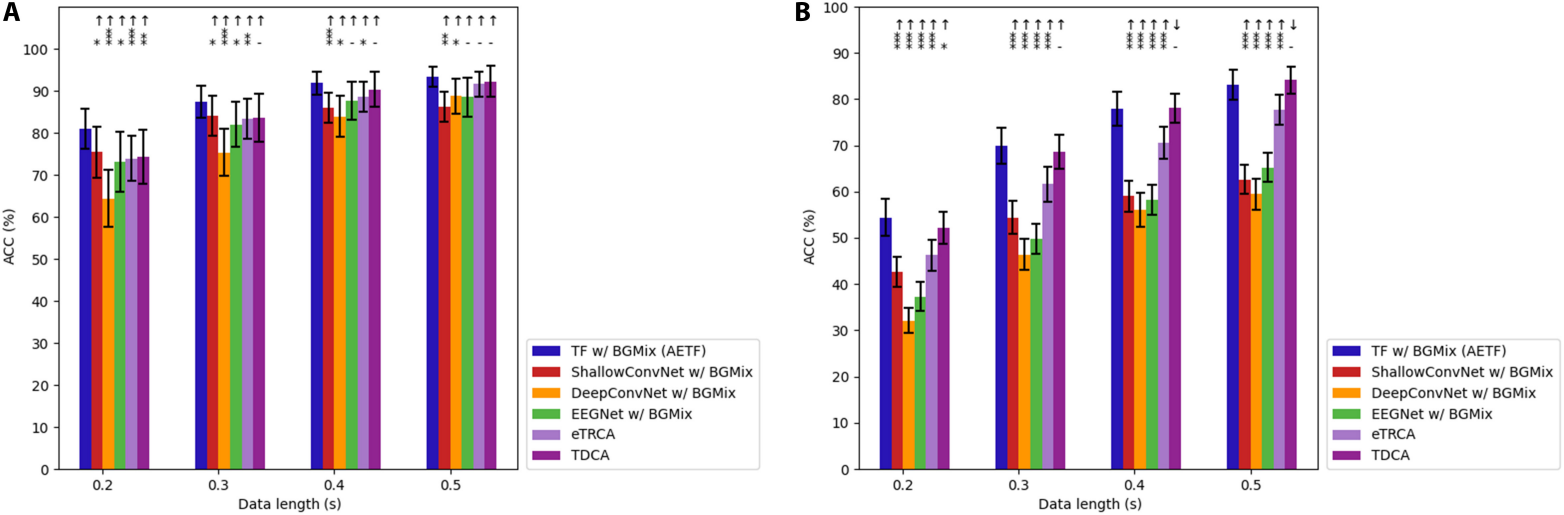
Accuracy comparison of 6 methods in 2 SSVEP datasets. (A) Dataset I. (B) Dataset II. For each baseline algorithm, a statistical test between the AETF and the baseline is conducted. The asterisks indicate the direction and significance of the increases (↑: positive increase, ↓: negative increase; *P < 0.05, **P < 0.01, ***P < 0.001). The error bars represent SEM.

Fig. 13 shows the classification accuracy of AETF and the baseline algorithms with different training data lengths. In dataset I, the AETF outperformed other baseline models in each case and achieved accuracies of 80.1%, 87.3%, 91.8%, and 93.2% with training data length *t* = 0.2, 0.3, 0.4, and 0.5 s, respectively. In dataset II, the AETF obtained accuracies of 54.1%, 69.2%, 77.3%, and 82.5% with *t* = 0.2, 0.3, 0.4, and 0.5. The classification result showed that the AETF with BGMix achieved the best performance in most cases, and its superiority was more pronounced under shorter training data length. Moreover, the performance of deep learning models in dataset I surpasses that in dataset II, and even EEGNet with BGMix achieves competitive results compared with eTRCA and TDCA. We attribute this to dataset I’s larger number of intra-class training samples, which ensures greater diversity in the data and the generated samples.

**Fig. 14.**
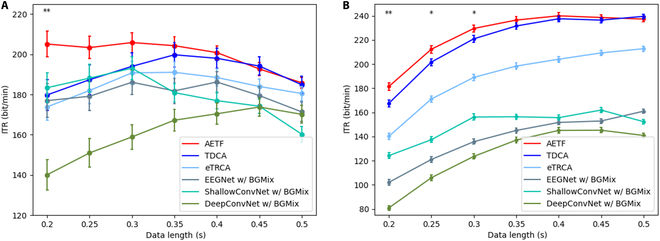
ITR performance comparison of AETF and TDCA in the public SSVEP datasets. (A) Dataset I. (B) Dataset II. The asterisks in the subfigures indicate significant difference between each pair of AETF and TDCA (*P < 0.05, **P < 0.01, ***P < 0.001). The error bars represent SEM.

To further illustrate the proposed model’s performance, we evaluated the ITR performance of the AETF and baseline models in both datasets. Moreover, the ITRs of AETF and TDCA were statistically compared under various training data lengths to validate the superior performance of AETF. Fig. 14 shows that the AETF achieved significant improvements under short training data lengths and achieved the highest ITR of 205.82 ± 15.81 bits/min and 240.03 ± 14.91 bits/min in datasets I and II, respectively.

### Visualization and interpretability

**Fig. 15.**
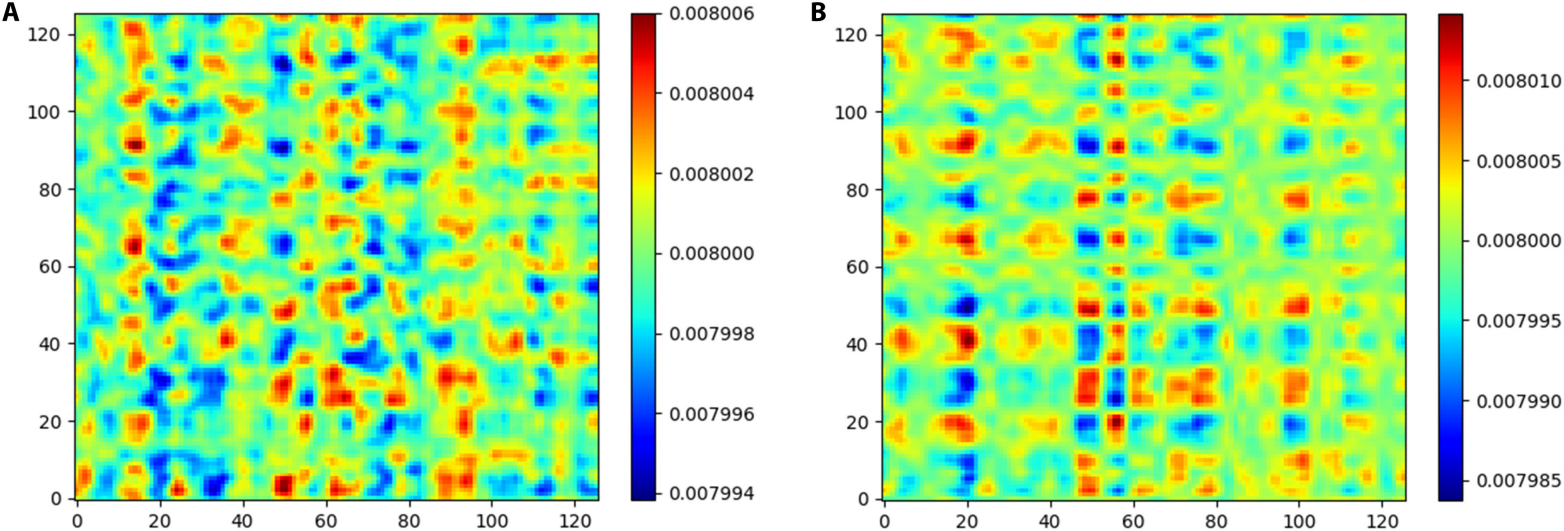
The attention matrix of of the Transformer modules. (A) First TF layer. (B) Second TF layer.

To analyze the working principles of the Transformer module, we visualized the self-attention of the 2-layer Transformer module, and an example chosen from dataset I appears in Fig. 15. Fig. 15A and B shows the attentions of the first and second layer, respectively. For a 0.5-s (125 sample points) SSVEP signal, the attention forms a 125 × 125 matrix, and the value $V_{a,b}$ of the matrix indicates the importance of point *b* to point *a*. The attention matrix highlights the vital temporal information along the sequence. For the first layer, the distribution of important features is irregular, which confirms the ability of Transformer to extract varied temporal features. In the second layer, after the feature processing of the first layer, the distribution of important features becomes more regular, and the vital features locate at fixed points. This result shows that the Transformer module successfully extracts and normalizes the vital temporal features.

**Fig. 16.**
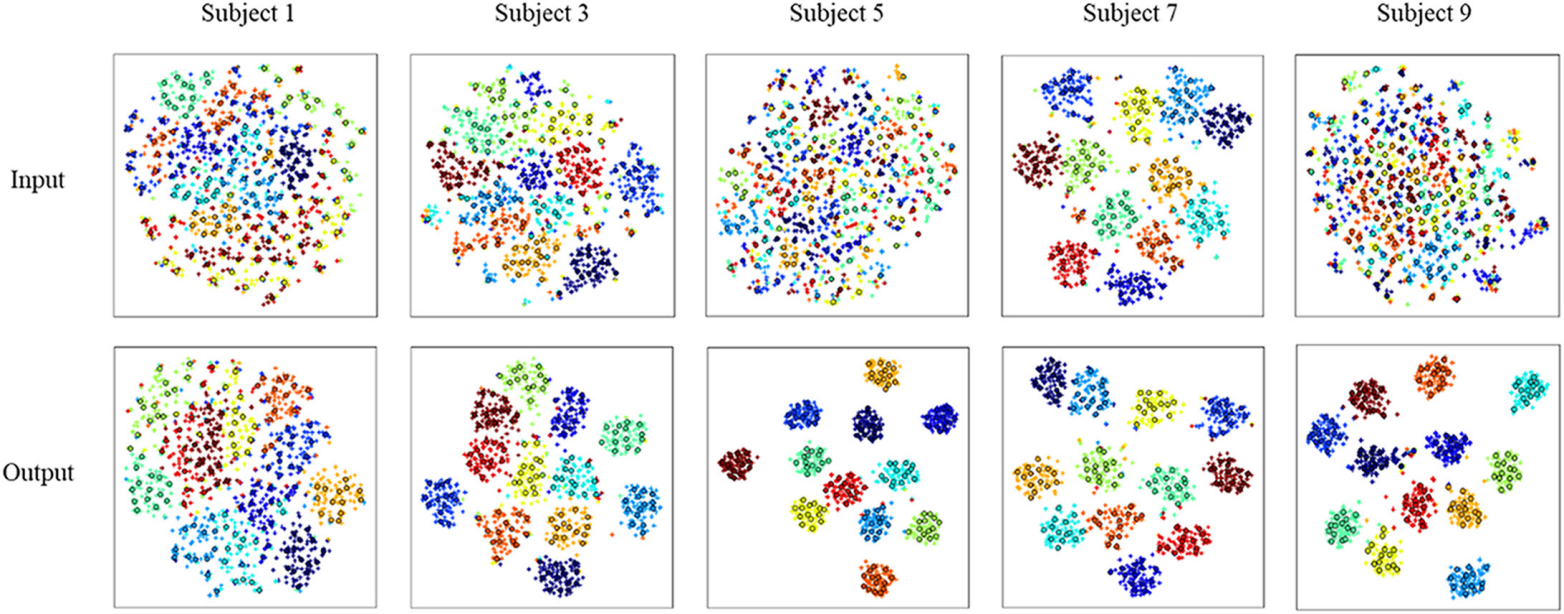
Visualization with *t*-SNE. The top subfigures show the distributions of the input SSVEP signal, while the bottom subfigures show the distributions of the TF module outputs. Dots represent training samples, and crosses represent generated samples.

To further assess the effectiveness of the BGMix strategy and the AETF model, we applied the *t*-distributed stochastic neighbor embedding (*t*-SNE) method to visualize the distribution of input samples, generated samples, and the corresponding output features of the AETF model. As illustrated in Fig. 16, we analyzed the data from 5 subjects in dataset I using *t*-SNE. The upper panels present the distribution of the input data, while the lower panels depict the high-dimensional outputs of the Transformer module. In each panel, training samples are represented as dots, generated samples as crosses, and the color of each sample indicates its class.

The results indicate that, for each subject, the distribution of generated samples closely matches with that of the training samples in both the input data and output features. Moreover, the AETF outputs demonstrate enhanced regularity and separability when compared to the input data. These findings confirm the effectiveness of the proposed methods.

## Discussion

This study addresses the persistent challenge of data sparsity in EEG research by introducing a novel data augmentation strategy, BGMix. Unlike traditional data augmentation techniques in CV, BGMix is rooted in the neural mechanisms underlying EEG signals, ensuring that the generated samples preserve the natural data distribution of SSVEP signals. This approach significantly improves the performance of various deep learning models. To fully leverage the abundant training samples generated by BGMix, we developed AETF to incorporate a Transformer module to extract complex temporal features effectively.

We validated the effectiveness of the proposed approach through classification experiments on 2 public SSVEP datasets. Results show that the AETF delivered significant improvements over baseline algorithms, recording the highest ITRs of 205.82 ± 15.81 bits/min and 240.03 ± 14.91 bits/min on the respective datasets. Crucially, the BGMix strategy played a key role in boosting the decoding efficiency of deep learning models for SSVEP classification.

### Addressing data sparsity with BGMix

Data sparsity poses a significant challenge in EEG-based BCI classification tasks, particularly for deep learning models. Unlike image or text data, EEG signals are inherently less interpretable, requiring augmentation strategies incorporating prior knowledge of EEG characteristics. The BGMix strategy leverages a fundamental property of EEG signals: the consistency of task-related components across trials of the same class and the variability in background noise. By blending task-related components with diverse background noise, BGMix generates samples that maintain the essential features of authentic EEG signals. Additionally, the BGMix strategy addresses not only the data sparsity issue but also the data imbalance issue across classes. By generating new data based on the characteristics of EEG signals, it ensures that the number of training samples per class remains balanced.

Compared to existing data augmentation methods, such as the sliding window [18], noise injection [19], and generative adversarial networks [20], the BGMix strategy offers a simple yet effective alternative with a clear electroencephalographic basis. Samples generated by the BGMix strategy largely retain the statistical distribution of the original data, further enhancing its applicability and reliability in EEG-based BCI tasks. Furthermore, the comparison between the BGMix and conventional data augmentation techniques demonstrates that, when applied to short-length training data, the BGMix excels in generating a large quantity of high-quality training samples with greater diversity.

**Fig. 17.**
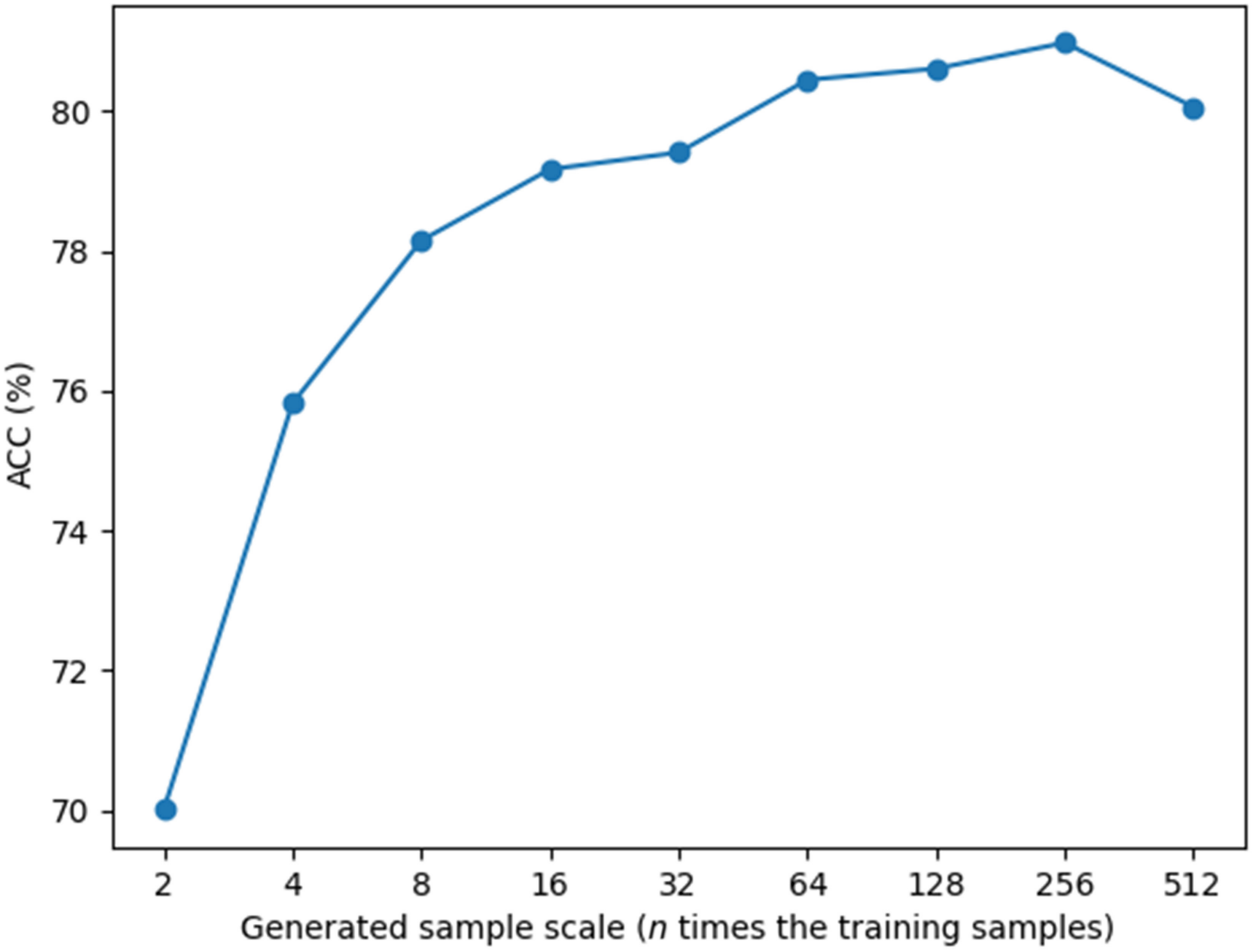
Accuracy of AETF with different amount of generated training samples.

We further evaluated the impact of augmented data by analyzing the classification accuracy of AETF on dataset I using varying quantities of training samples generated by BGMix. As illustrated in Fig. 17, accuracy increased with more generated data, stabilizing after approximately 64 times the original training sample size. However, performance declined beyond a certain threshold (256 times the original sample size), likely because of limited data diversity in finite training samples. This finding highlights the diminishing returns of augmentation and underscores the importance of optimizing the amount of generated data—a process requiring empirical tuning and contextual adjustments. Future studies could explore combining BGMix with other augmentation strategies or leveraging transfer learning methods to further address data sparsity challenges.

### Improving decoding efficiency by using Transformer module

In recent years, researchers have widely adopted Transformers in fields such as CV and NLP, where they have demonstrated exceptional performance in extracting high-dimensional features and managing long-range dependencies within sequences. These characteristics make Transformers particularly suitable for EEG signal processing, as EEG data often involve complex spatiotemporal patterns and long-duration recordings. The results of this study indicate that the effective application of Transformers depends heavily on diverse and extensive datasets, aligning well with data augmentation techniques designed to enhance the variability and richness of EEG data. By integrating data augmentation strategies with a Transformer-based framework, researchers can develop versatile and robust approaches for decoding EEG signals, enabling broader applications across various EEG-related tasks.

### Comparison with state-of-the-art methods

Traditional decomposition methods, such as eTRCA and TDCA, have demonstrated strong performance in SSVEP classification. For instance, TDCA achieved competitive ITR scores in this study, although these scores were slightly lower than those reported in reference studies—potentially because of differences in preprocessing or accuracy calculations. Nonetheless, the AETF with BGMix consistently outperformed these methods in scenarios with limited training data length, delivering faster training times and improving BCI system efficiency.

Compared to deep learning approaches, the CNN-based network proposed by Guney et al. [47] achieved ITRs comparable to those of the AETF on dataset II. However, their method did not include a validation set and early-stopping mechanisms, which weakened the model’s generalizability and applicability in real-world settings.

To the best of the authors’ knowledge, no existing deep learning model has outperformed the AETF framework in SSVEP classification, particularly under conditions involving short training data lengths. We attribute this superior performance to the collaborative effect of the AETF model and the BGMix data augmentation strategy. Specifically, this combination represents an effective alignment between the increased diversity provided by augmented data and the representational capacity of the model. As demonstrated in Performance improvement with BGMix strategy, the TF model does not surpass other deep learning models when trained without BGMix. However, when BGMix is applied, the same model achieves state-of-the-art performance, highlighting the importance of this integrated approach.

The success of our method in short-training-duration scenarios is grounded in the presence of stable and repeatable patterns in SSVEP signals. This characteristic forms the foundation of the BGMix strategy, which generates new samples by introducing diverse background noise. This augmentation improves the model’s ability to generalize and enhances its robustness to non-task-related neural fluctuations. Furthermore, as the training data length increases, the classification performance of all models tends to converge toward a high level of accuracy. In such cases, the relative differences in decoding performance diminish, suggesting that the advantages of the AETF framework are particularly pronounced in short-training-duration scenarios where robust generalization is more difficult to achieve.

### Computational cost and application

**Fig. 18.**
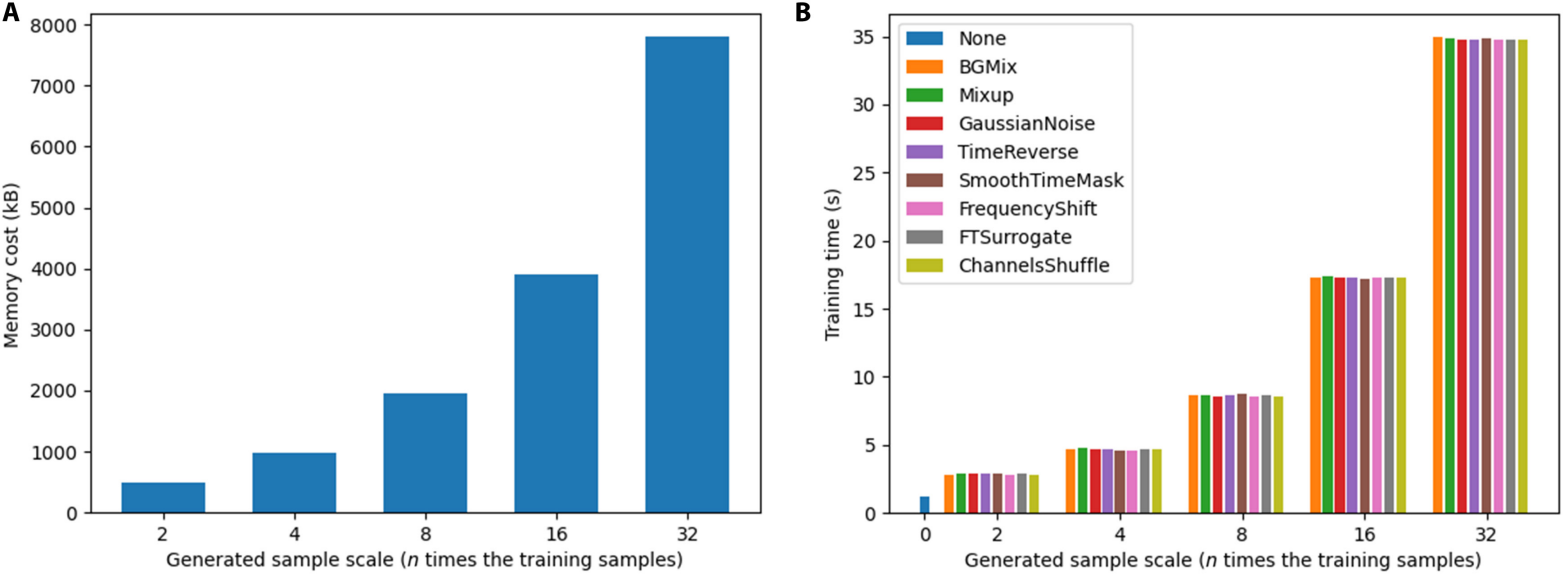
Computional cost of of the AETF. (A) Memory cost for data augmentation approaches. (B) Training time of TF model using various data augmentation approaches.

The ultimate objective of BCI decoding algorithms is their deployment in real-world applications, where they can serve as viable alternatives for human–computer interaction. To this end, it is crucial to evaluate the computational efficiency of these algorithms to ensure their engineering feasibility. We first assessed the memory consumption associated with training samples of 0.2 s from a single subject in dataset I. As shown in Fig. 18A, memory usage across all data augmentation methods remained at the same level, which increased exponentially along with the number of generated samples. Subsequently, we examined the training time of the TF model using different data augmentation strategies over 100 training epochs for one subject in a single fold. The results, presented in Fig. 18B, indicate that training time follows a similar trend—remaining stable across augmentation methods but growing proportionally with the volume of augmented data. These findings align with intuitive expectations and confirm that the computational cost of the proposed BGMix method is comparable to that of conventional data augmentation techniques.

We then evaluated model efficiency across baseline methods, with results summarized in Table 2. For eTRCA and TDCA, the number of spatial filter weights is recorded as the model’s parameters. For deep learning methods, the parameters refer to the trainable weights specific to each model. Meanwhile, the memory cost is measured using the memory_allocated function from the PyTorch library, which is applicable exclusively to deep learning models. Among all models, AETF exhibits the highest parameter count and memory usage. This, coupled with the substantial number of generated training samples and the complexity of its attention mechanism, results in a significantly longer training time for AETF. To address this limitation, we explored the application of model compression techniques to reduce the computational burden of AETF, specifically through knowledge distillation.

**Table 2.** Computional cost of models

Model	Parameter amount	Memory allocated/MB	Training time per fold/s
eTRCA	96	N/A	0.6388
TDCA	2,500	N/A	0.8687
ShallowConvNet	16,892	16.77	2.323
DeepConvNet	181,432	19.42	3.0576
EEGNet	18,060	18.32	3.7737
TF	186,288	25.14	5.4507
AETF_1layer	139,296	21.69	3.6442

Knowledge distillation is a widely adopted technique in which a smaller, computationally efficient model (referred to as the student) is trained to replicate the behavior of a larger, more complex model (the teacher) [48]. In this study, the original AETF model served as the teacher, while a simplified student variant—designated AETF_1layer—was constructed using a single Transformer encoder layer and a reduced hidden dimension size (halved relative to the teacher). The student model was trained using a combination of hard labels (i.e., ground-truth class annotations) and soft targets produced by the teacher model. This dual-supervision approach enables the student to learn not only the correct classifications but also the probabilistic output distributions shaped by the teacher’s learned knowledge.

To improve the informativeness of the soft targets, a temperature scaling factor of 5 was applied to the teacher’s output logits, which smooths the predicted probability distribution and highlights interclass similarities. During training, the total loss was computed as a weighted combination of the hard loss and soft loss, with a fixed ratio of 0.7:0.3, respectively. This configuration was chosen to balance fidelity to the true labels with guidance from the teacher model’s predictive behavior. For further methodological details, including architectural specifications and training protocols, please refer to Ref. [48].

**Fig. 19.**
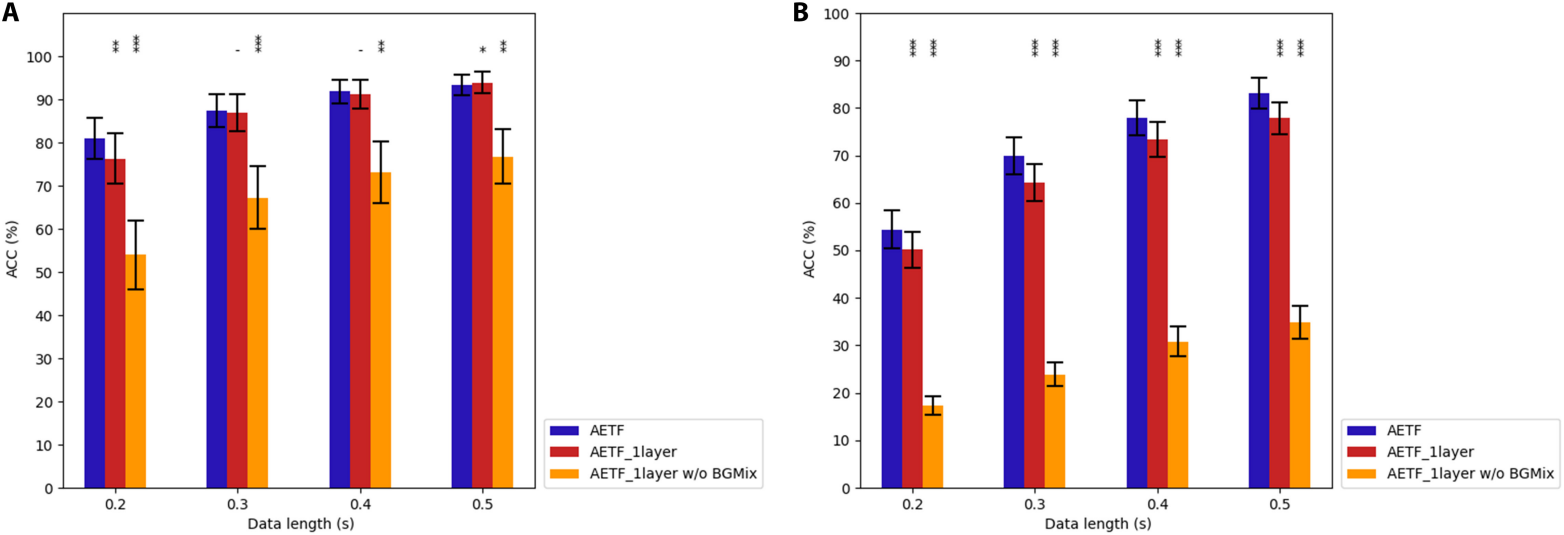
Accuracy comparison between AETF and distilled models. (A) Dataset I. (B) Dataset II.The asterisks indicate significant difference between the AETF and distilled models (*P < 0.05, **P < 0.01, ***P < 0.001). The error bars represent SEM.

As illustrated in Fig. 19A and B, AETF_1layer achieved comparable performance to the original AETF model, with only minor reductions observed—an average accuracy drop of 1.27% on dataset I and 4.92% on dataset II. Moreover, as shown in Table 1, the reduced model size resulted in lower memory usage and faster training times, demonstrating the practical feasibility of using knowledge distillation for model compression. However, when the BGMix strategy was not employed, the performance of AETF_1layer deteriorated substantially, with average accuracy reductions of 20.59% and 25.7% in datasets I and II, respectively. These results underscore the synergistic relationship between the BGMix augmentation strategy and Transformer-based architectures, highlighting the necessity of their combined use for optimal performance.

In real-world application scenarios, the efficiency of EEG decoding is not only determined by algorithmic performance but also significantly influenced by human factors. For example, prior studies have demonstrated that a subject’s attention level can markedly impact BCI performance by affecting the quality of EEG signals [49,50]. Specifically, reduced attention leads to increased variation in the recorded signals, thereby diminishing data separability and ultimately impairing the performance of decoding models, including AETF. Moreover, low attention contributes to a higher incidence of invalid data, which in turn reduces the diversity of usable training samples. This limitation may directly constrains the effectiveness of the BGMix strategy, as the quality of generated synthetic samples is dependent on the representativeness and variability of the original dataset. Besides, experimental design factors such as experiment duration [51], visual comfort [52], and cognitive load [53] can also significantly affect user performance and system reliability under practical conditions.

To mitigate the potential adverse effects caused by various factors, it is imperative to develop more generalizable and robust models for EEG-BCI systems. Broadly speaking, machine learning techniques, including deep learning, are designed to construct robust models capable of recognizing patterns or controlling systems in real-world environments, despite the presence of various adverse factors and uncertainties [54–58]. In this regard, the integration of the BGMix data augmentation strategy with the AETF architecture presents a compelling approach. This combination leverages the complementary strengths of synthetic data diversity and the advanced representational capacity of a sophisticated model. This integrative framework offers a promising direction for future research in EEG-based BCI, particularly in scenarios characterized by low data availability and high variability.

### Limitations and future directions

While the AETF outperforms existing models, several limitations persist. Deep learning models for EEG classification typically require longer training times and higher computational resources than traditional matrix decomposition algorithms, posing challenges for real-time BCI deployment. The complexity of TF models and their reliance on large datasets further exacerbate these issues. Additionally, a notable limitation of the BGMix strategy is that it can only be effectively implemented in conjunction with deep learning models. This constraint arises from the fact that BGMix is a variant of the Mixup strategy, which generates synthetic samples associated with a pair of distinct labels. These mixed labels are then incorporated into the training process via a composite loss function—typically involving a weighted combination of 2 separate loss terms—a mechanism that is inherently supported by the flexible optimization framework of deep learning architectures. Such an approach is not readily compatible with traditional machine learning models, which lack the necessary infrastructure to process soft label assignments and mixed-objective loss functions. This limitation highlights a promising avenue for future work: the development of novel data augmentation techniques utilizing background EEG components that are compatible with both deep learning and classical machine learning paradigms.

This study also identified an optimal threshold for augmented data volume, beyond which additional data yield diminishing returns. This highlights the importance of careful hyperparameter tuning when setting augmentation ratios, as these depend on specific application scenarios and empirical knowledge. Future work could investigate adaptive augmentation methods that dynamically adjust ratios based on dataset size and model performance.

Additionally, while the AETF leverages a standard Transformer architecture, emerging Transformer variants tailored for EEG signal processing (e.g. [59,60]) may enhance its performance further. Incorporating multimodal and transfer learning techniques may also improve classification accuracy and robustness, paving the way for more versatile and reliable EEG-based BCI systems.

## Data Availability

The SSVEP datasets used in this study are publicly available and can be accessed from the following websites (dataset I: https://github.com/mnakanishi/12JFPM_SSVEP/tree/master/data; dataset II: [38]). The source code is available at https://github.com/ccc65535/AETF.

## Supplementary Materials

Supplementary 1 Algorithm S1 Tables S1 to S4
