# Supplementary material for: Augmenting Electroencephalogram Transformer for Steady-State Visually Evoked Potential-Based Brain–Computer Interfaces
Source: Cyborg Bionic Syst. 2025 Oct 7;6:0379. doi: 10.34133/cbsystems.0379 (PMC12501431; doi:10.34133/cbsystems.0379)
Supplement: Supplementary 1 — Algorithm S1 Tables S1 to S4 [file cbsystems.0379.f1.pdf]

# Supplementary Materials

**Algorithm 1** Mix function of the BGMix

```
# Input
## block_a, block_b: 2 blocks of data from different classes
## nt : n trials selected to compute averaged template

# Output
## build_block_a,build_block_b: generated samples

def block_mix(block_a, block_b, nt=2):
    nTrials, nChan, nSamp = block_a.shape

    # shuffle the trials
    rnd_ind = [i for i in range(nTrials)]
    random.shuffle(rnd_ind)
    block_a = block_a[rnd_ind,:,:]
    random.shuffle(rnd_ind)
    block_b = block_b[rnd_ind,:,:]

    build_block_a = np.zeros([nTrials,nChan,nSamp])
    build_block_b = np.zeros([nTrials,nChan,nSamp])

    # choose nt trials to generate new sample
    for ns in range(0, nTrials, nt):
        trials=[i%nTrials for i in range(ns,ns+nt)]

        trials_a=block_a[trials,:,:]
        trials_b=block_b[trials,:,:]

        template_a=np.mean(trials_a,axis=0,keepdims=True)
                                        .repeat(nt,0)
        template_b=np.mean(trials_b,axis=0,keepdims=True)
                                        .repeat(nt,0)

        bg_a=trials_a-template_a
        bg_b=trials_b-template_b

        gen_a=template_a+bg_b
        gen_b=template_b+bg_a

        build_block_a[trials,:,:]=gen_a
        build_block_b[trials,:,:]=gen_b
    return build_block_a,build_block_b
```

Table S1: Subject-specific accuracy improvement with BGMix in Dataset I

| Subject No. | Shallow-ConvNet | ShallowConvNet w/ BGMix | Improve-ment | Deep-ConvNet | DeepConvNet w/ BGMix | Improve-ment |
|---|---|---|---|---|---|---|
| 1 | 0.327 | 0.790 | 0.463 | 0.384 | 0.529 | 0.145 |
| 2 | 0.235 | 0.436 | 0.201 | 0.139 | 0.353 | 0.214 |
| 3 | 0.455 | 0.851 | 0.396 | 0.421 | 0.639 | 0.218 |
| 4 | 0.696 | 0.913 | 0.217 | 0.718 | 0.892 | 0.174 |
| 5 | 0.554 | 0.876 | 0.322 | 0.621 | 0.800 | 0.179 |
| 6 | 0.834 | 0.941 | 0.107 | 0.774 | 0.905 | 0.131 |
| 7 | 0.785 | 0.891 | 0.106 | 0.663 | 0.771 | 0.108 |
| 8 | 0.680 | 0.847 | 0.167 | 0.609 | 0.798 | 0.189 |
| 9 | 0.297 | 0.618 | 0.321 | 0.323 | 0.502 | 0.179 |
| 10 | 0.218 | 0.377 | 0.159 | 0.154 | 0.251 | 0.097 |
| Avg. | 0.508 | 0.754 | 0.246 | 0.481 | 0.644 | 0.163 |

Table S2: Subject-specific accuracy improvement with BGMix in Dataset I

| Subject No. | EEGNet | EEGNet w/ BGMix | Improve-ment | TF | AETF | Improve-ment |
|---|---|---|---|---|---|---|
| 1 | 0.48 | 0.763 | 0.283 | 0.613 | 0.806 | 0.193 |
| 2 | 0.316 | 0.406 | 0.090 | 0.283 | 0.632 | 0.348 |
| 3 | 0.592 | 0.836 | 0.244 | 0.689 | 0.890 | 0.200 |
| 4 | 0.764 | 0.929 | 0.165 | 0.761 | 0.912 | 0.150 |
| 5 | 0.687 | 0.789 | 0.102 | 0.461 | 0.907 | 0.446 |
| 6 | 0.864 | 0.951 | 0.087 | 0.870 | 0.962 | 0.093 |
| 7 | 0.827 | 0.929 | 0.102 | 0.853 | 0.925 | 0.072 |
| 8 | 0.753 | 0.87 | 0.117 | 0.779 | 0.885 | 0.106 |
| 9 | 0.377 | 0.576 | 0.199 | 0.435 | 0.715 | 0.280 |
| 10 | 0.267 | 0.259 | -0.008 | 0.355 | 0.467 | 0.112 |
| Avg. | 0.593 | 0.731 | 0.138 | 0.610 | 0.810 | 0.200 |

Table S3: Subject-specific accuracy improvement with BGMix in Dataset II

| Subject No. | Shallow-ConvNet | ShallowConvNet w/ BGMix | Improve-ment | Deep-ConvNet | DeepConvNet w/ BGMix | Improve-ment |
|---|---|---|---|---|---|---|
| 1 | 0.249 | 0.608 | 0.360 | 0.271 | 0.389 | 0.118 |
| 2 | 0.176 | 0.500 | 0.324 | 0.140 | 0.320 | 0.180 |
| 3 | 0.501 | 0.876 | 0.375 | 0.289 | 0.731 | 0.441 |
| 4 | 0.394 | 0.764 | 0.370 | 0.283 | 0.666 | 0.384 |
| 5 | 0.149 | 0.460 | 0.311 | 0.157 | 0.317 | 0.160 |
| 6 | 0.232 | 0.331 | 0.100 | 0.196 | 0.236 | 0.040 |
| 7 | 0.164 | 0.405 | 0.241 | 0.140 | 0.301 | 0.160 |
| 8 | 0.155 | 0.359 | 0.204 | 0.195 | 0.278 | 0.083 |
| 9 | 0.202 | 0.365 | 0.163 | 0.193 | 0.197 | 0.004 |
| 10 | 0.143 | 0.299 | 0.156 | 0.108 | 0.151 | 0.043 |
| 11 | 0.031 | 0.105 | 0.074 | 0.025 | 0.078 | 0.053 |
| 12 | 0.055 | 0.189 | 0.133 | 0.063 | 0.160 | 0.097 |
| 13 | 0.069 | 0.421 | 0.351 | 0.082 | 0.217 | 0.134 |
| 14 | 0.271 | 0.543 | 0.272 | 0.207 | 0.386 | 0.180 |
| 15 | 0.137 | 0.350 | 0.213 | 0.101 | 0.225 | 0.124 |
| 16 | 0.134 | 0.307 | 0.173 | 0.110 | 0.297 | 0.186 |
| 17 | 0.210 | 0.478 | 0.268 | 0.189 | 0.350 | 0.161 |
| 18 | 0.395 | 0.625 | 0.231 | 0.404 | 0.466 | 0.062 |
| 19 | 0.068 | 0.073 | 0.005 | 0.062 | 0.044 | -0.018 |
| 20 | 0.126 | 0.288 | 0.162 | 0.144 | 0.222 | 0.078 |
| 21 | 0.137 | 0.270 | 0.133 | 0.112 | 0.249 | 0.137 |
| 22 | 0.377 | 0.650 | 0.274 | 0.336 | 0.550 | 0.214 |
| 23 | 0.134 | 0.370 | 0.235 | 0.202 | 0.236 | 0.034 |
| 24 | 0.234 | 0.510 | 0.276 | 0.259 | 0.325 | 0.066 |
| 25 | 0.141 | 0.448 | 0.307 | 0.226 | 0.326 | 0.100 |
| 26 | 0.189 | 0.508 | 0.319 | 0.227 | 0.481 | 0.254 |
| 27 | 0.189 | 0.520 | 0.330 | 0.221 | 0.396 | 0.175 |
| 28 | 0.230 | 0.525 | 0.295 | 0.198 | 0.416 | 0.217 |
| 29 | 0.115 | 0.203 | 0.087 | 0.036 | 0.152 | 0.116 |
| 30 | 0.170 | 0.400 | 0.230 | 0.190 | 0.339 | 0.149 |
| 31 | 0.353 | 0.841 | 0.488 | 0.338 | 0.653 | 0.315 |
| 32 | 0.352 | 0.598 | 0.245 | 0.302 | 0.534 | 0.232 |
| 33 | 0.075 | 0.175 | 0.100 | 0.042 | 0.119 | 0.077 |
| 34 | 0.183 | 0.427 | 0.244 | 0.213 | 0.309 | 0.096 |
| 35 | 0.096 | 0.127 | 0.031 | 0.093 | 0.125 | 0.032 |
| Avg. | 0.195 | 0.426 | 0.231 | 0.182 | 0.321 | 0.140 |

Table S4: Subject-specific accuracy improvement with BGMix in Dataset II

| Subject No. | EEGNet | EEGNet w/ BGMix | Improvement | TF | AETF | Improvement |
|---|---|---|---|---|---|---|
| 1 | 0.453 | 0.502 | 0.049 | 0.535 | 0.826 | 0.291 |
| 2 | 0.325 | 0.353 | 0.028 | 0.438 | 0.684 | 0.246 |
| 3 | 0.711 | 0.764 | 0.053 | 0.757 | 0.938 | 0.181 |
| 4 | 0.575 | 0.699 | 0.124 | 0.485 | 0.914 | 0.429 |
| 5 | 0.242 | 0.311 | 0.069 | 0.339 | 0.709 | 0.370 |
| 6 | 0.292 | 0.376 | 0.084 | 0.329 | 0.334 | 0.006 |
| 7 | 0.259 | 0.262 | 0.003 | 0.314 | 0.534 | 0.219 |
| 8 | 0.359 | 0.381 | 0.022 | 0.319 | 0.567 | 0.249 |
| 9 | 0.246 | 0.262 | 0.016 | 0.341 | 0.419 | 0.078 |
| 10 | 0.277 | 0.255 | -0.022 | 0.333 | 0.562 | 0.228 |
| 11 | 0.067 | 0.053 | -0.014 | 0.050 | 0.109 | 0.059 |
| 12 | 0.16 | 0.165 | 0.005 | 0.099 | 0.362 | 0.264 |
| 13 | 0.177 | 0.227 | 0.050 | 0.130 | 0.521 | 0.391 |
| 14 | 0.451 | 0.509 | 0.058 | 0.333 | 0.698 | 0.365 |
| 15 | 0.254 | 0.320 | 0.066 | 0.268 | 0.447 | 0.178 |
| 16 | 0.236 | 0.273 | 0.037 | 0.222 | 0.307 | 0.085 |
| 17 | 0.359 | 0.423 | 0.064 | 0.390 | 0.532 | 0.141 |
| 18 | 0.53 | 0.577 | 0.047 | 0.538 | 0.713 | 0.175 |
| 19 | 0.106 | 0.080 | -0.026 | 0.060 | 0.088 | 0.027 |
| 20 | 0.226 | 0.232 | 0.006 | 0.271 | 0.309 | 0.038 |
| 21 | 0.221 | 0.299 | 0.078 | 0.188 | 0.255 | 0.068 |
| 22 | 0.549 | 0.621 | 0.072 | 0.507 | 0.819 | 0.312 |
| 23 | 0.278 | 0.265 | -0.013 | 0.217 | 0.515 | 0.298 |
| 24 | 0.45 | 0.524 | 0.074 | 0.328 | 0.574 | 0.245 |
| 25 | 0.375 | 0.466 | 0.091 | 0.183 | 0.489 | 0.306 |
| 26 | 0.378 | 0.481 | 0.103 | 0.166 | 0.586 | 0.420 |
| 27 | 0.389 | 0.431 | 0.042 | 0.264 | 0.733 | 0.469 |
| 28 | 0.332 | 0.392 | 0.060 | 0.436 | 0.745 | 0.309 |
| 29 | 0.151 | 0.167 | 0.016 | 0.141 | 0.309 | 0.168 |
| 30 | 0.235 | 0.301 | 0.066 | 0.289 | 0.546 | 0.257 |
| 31 | 0.732 | 0.811 | 0.079 | 0.509 | 0.953 | 0.444 |
| 32 | 0.555 | 0.602 | 0.047 | 0.592 | 0.830 | 0.239 |
| 33 | 0.111 | 0.134 | 0.023 | 0.135 | 0.181 | 0.046 |
| 34 | 0.418 | 0.401 | -0.017 | 0.329 | 0.617 | 0.287 |
| 35 | 0.244 | 0.136 | -0.108 | 0.147 | 0.216 | 0.069 |
| Avg. | 0.335 | 0.373 | 0.038 | 0.314 | 0.541 | 0.227 |
